# The Cytotoxic Natural Product Vioprolide A Targets Nucleolar Protein 14, Which Is Essential for Ribosome Biogenesis

**DOI:** 10.1002/anie.201911158

**Published:** 2019-12-12

**Authors:** Volker C. Kirsch, Christina Orgler, Simone Braig, Irmela Jeremias, David Auerbach, Rolf Müller, Angelika M. Vollmar, Stephan A. Sieber

**Affiliations:** ^1^ Center for Integrated Protein Science (CIPSM) Department Chemie Technische Universität München (TUM) Lichtenbergstrasse 4 85747 Garching Germany; ^2^ Department of Pharmacy Pharmaceutical Biology Ludwig-Maximilian-University of Munich (LMU) Butenandtstrasse 5–13 81377 Munich Germany; ^3^ Research Unit Apoptosis in Hematopoietic Stem Cells Helmholtz Zentrum München German Research Center for Environmental Health Marchioninistrasse 25 81377 München Germany; ^4^ Dr. von Hauner Children's Hospital Ludiwg-Maximilian-University of Munich (LMU) Lindwurmstrasse 4 80337 Munich Germany; ^5^ Helmholtz Institute for Pharmaceutical Research Saarland (HIPS) Helmholtz Center for Infection Research and Department of Pharmacy Saarland University Campus Building E8.1 66123 Saarbrücken Germany

**Keywords:** acute lymphoblastic leukemia, natural products, quantitative proteomics, ribosomess, target identification

## Abstract

Novel targets are needed for treatment of devastating diseases such as cancer. For decades, natural products have guided innovative therapies by addressing diverse pathways. Inspired by the potent cytotoxic bioactivity of myxobacterial vioprolides A–D, we performed in‐depth studies on their mode of action. Based on its prominent potency against human acute lymphoblastic leukemia (ALL) cells, we conducted thermal proteome profiling (TPP) and deciphered the target proteins of the most active derivative vioprolide A (VioA) in Jurkat cells. Nucleolar protein 14 (NOP14), which is essential in ribosome biogenesis, was confirmed as a specific target of VioA by a suite of proteomic and biological follow‐up experiments. Given its activity against ALL cells compared to healthy lymphocytes, VioA exhibits unique therapeutic potential for anticancer therapy through a novel mode of action.

## Graphical abstract

**On target**: Functional characterization of VioA revealed potent growth inhibitory effects in cancer cells and remarkable apoptosis induction in acute lymphoblastic leukemia cells, without affecting healthy peripheral blood mononuclear cells. Thermal proteome profiling studies revealed nucleolar protein 14 (NOP14) as a protein target, which was further validated using proteomics and biological follow‐up experiments.

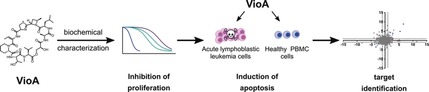

Natural products display a rich source of bioactive molecules selected by evolution to target distinct molecular pathways in eukaryotic and prokaryotic cells.^1^ One of the preeminent producers of a wide range of bioactive secondary metabolites are myxobacteria.^2^ In 1996, the isolation of peptolides vioprolides A–D (Figure 1 A) was reported from *Cystobacter violaceus* and detailed studies on their biosynthesis have recently been performed.^3^ Despite their antifungal and anticancer activities in the low nanomolar range, as well as immunomodulatory activity in micromolar concentrations,^4^ no molecular target elucidation has been reported up to date.^5^

**Figure 1:**
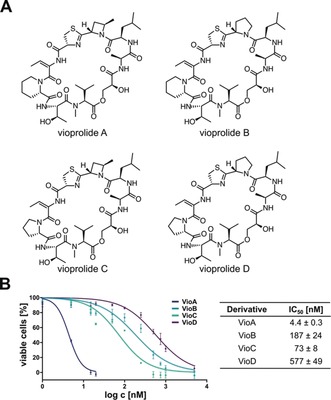
Dose‐dependent inhibition of leukemia cell proliferation by vioprolide derivatives. A) Structures of vioprolides A, B, C and D. B) Dose–response curves and corresponding IC_50_ values from Jurkat cells incubated with vioprolides A, B, C or D, as determined by cell titer blue assay after 72 h. Data points represent the mean±SEM of three independent experiments performed in triplicate.

Despite the wealth of target identification methods available, including activity‐based protein profiling (ABPP)^6^, ^7^ and thermal proteome profiling (TPP),^8^, ^9^ numerous natural products still lack functional characterization. This information is needed in order to decipher novel and unprecedented modes of action in cancer cells, where drug resistance, therapeutic failure, or relapse require new targets and cellular pathways with high specificity towards tumor cells.^10^

By applying diverse proteomic and biochemical methods we investigated the cellular mechanism of vioprolide A (VioA) in Jurkat cancer cells and recovered nucleolar protein 14 (NOP14), which is essential for ribosome biogenesis, to be a specific target.

Prior to target‐identification studies, we ranked vioprolide A–D potencies in the acute lymphoblastic leukemia (ALL) cell line Jurkat to select the most potent growth inhibitory compound for subsequent mode of action analysis. VioA exhibited the highest antiproliferative activity, with a half maximal inhibitory concentration (IC_50_) of 4 nm (Figure 1 B). In line with a previous report,^5^ the vioprolides showed significant differences in bioactivity, thus demonstrating that the structural composition of the N‐heterocycles is crucial for potency (Figure 1 A).

Moreover, VioA displayed low nanomolar IC_50_ values in different cell lines, thus illustrating its broad potency (Figure 2 A, Figure S1 in the Supporting Information). To analyze whether enhanced cell death rates contribute to diminished proliferative capacity of VioA‐treated cancer cells, apoptosis assays were performed. VioA exhibited an EC_50_ of 15.8 and 28.9 nm in CEM and Jurkat cells, respectively, signifying a high apoptosis rate in acute lymphoblastic leukemia (ALL) cells, compared to the acute myeloid leukemia (AML) cell line HL‐60 (EC_50_: 88.1 nm), as well as HeLa (EC_50_: 134 nm) and T24 cells (EC_50_: n.a.; Figure 2 B). This was confirmed by the extent of caspase‐3 activation and PARP cleavage, as analyzed by western blotting (Figure S2). Mechanistic studies on apoptosis induction revealed intrinsic apoptotic cell death triggered by VioA (Figure S3, see discussion in the Supporting Information). Moreover, cell‐cycle progression in Jurkat cells treated with VioA showed a significant decrease in cells in S‐phase, as measured by flow cytometry analysis (Figure 2 C).

**Figure 2:**
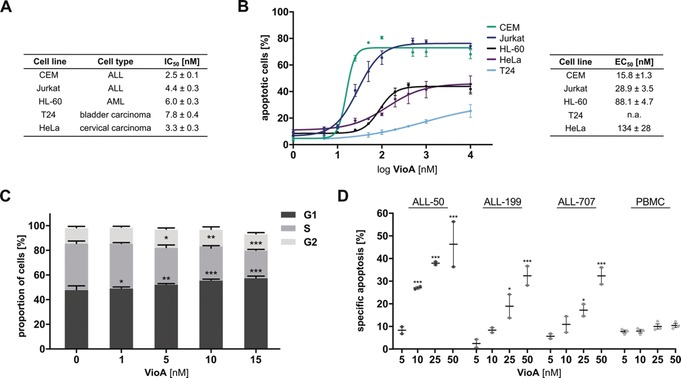
Anticancer effects of VioA. A) Inhibition of proliferation and corresponding IC_50_ values were analyzed by cell titer blue assay (suspension cell lines) or crystal violet staining (adherent cell lines) after 72 h. Experiments were conducted in three independent experiments and performed in triplicates. B) Apoptosis of various cancer cell lines treated with VioA for 24 h. Percentage of apoptotic cells and corresponding EC_50_ values were determined by propidium iodide (PI) staining and flow cytometry. Data points always represent the mean±SEM of three independent experiments performed in triplicate. C) Cell‐cycle analysis of VioA‐treated Jurkat cells (24 h) was performed by PI staining and flow cytometry analysis. Bars show the mean±SEM of three independent experiments performed in triplicate, one‐way ANOVA, Dunnett's test, * *P*<0.033, ** *P*<0.002, *** *P*<0.001. D) ALL PDX cells and PBMCs treated with VioA for 24 h. Percentage of apoptotic cells was determined by flow cytometry and specific apoptosis was calculated relative to DMSO control. Data points represent mean±SEM of independent experiments performed in triplicates (PDX cells: *n*=2, PBMCs: *n*=3), one‐way ANOVA Dunnett's test, * *P*<0.033, *** *P*<0.001.

To confirm the potency of VioA in ALL and to analyze its putative therapeutic relevance, the compound was tested in ALL patient‐derived xenograft (PDX) cells. VioA‐treated ALL samples of diverse background (Table S1) exhibited an increased apoptosis rate after 24 h treatment, as shown by flow cytometry (Figure 2 D). Notably, peripheral blood mononuclear cells (PBMCs) of healthy donors barely responded to VioA compared to PDX samples (Figure 2 D), thus clearly demonstrating that VioA exhibits auspicious antileukemic properties.

To rationalize the molecular mechanism underlying the potency of VioA in ALL, we performed target identification through affinity based protein profiling (AfBPP).^7^, ^11^ The VioA scaffold was functionalized with a photocrosslinker and a biorthogonal linker to yield the probe VioA‐P (Scheme S1). Although the antiproliferative potency of the probe against Jurkat cells only dropped by 4‐fold (Figure S4), in situ labeling with concentrations ranging from 0.5 μM to 2 μM revealed known background binders^12^ among the enriched proteins, but no clear target (Figure S5). Since the modification with the minimal linker could have perturbed the binding properties to the dedicated target(s) and the probe ester bond suffered from limited stability (Figure S6), TPP was selected as an alternative modification‐free strategy.

Jurkat cells were incubated in situ with VioA (1 μm) or DMSO and subsequently exposed to a range of temperatures (37 °C–67 °C). Cells were lysed and soluble proteins were isolated by ultracentrifugation, tryptically digested, and labeled using isobaric tandem mass tag labels (TMT).^12^ Respective labeled samples were combined, subjected to hydrophilic‐interaction chromatography (HILIC) fractionation and finally analyzed by LC–MS/MS (Figure 3 A). *T*_m_ shifts were calculated from two replicates of VioA versus DMSO treatment and visualized after filtering (Figure 3 B; see the Supporting Information for filtering criteria). 67 proteins revealed a minimum temperature shift of 1 °C in both VioA‐ versus DMSO‐treated replicates and were manually inspected for proper melting curves. Six proteins (blue dots Figure 3 B; for melting curves, see Figure S7, Table S2) passed additional significance thresholds (see the Supporting Information). Among these, U2 snRNP‐associated SURP motif‐containing protein (U2SURP) and nucleolar protein 14 (NOP14) were highly stabilized upon VioA treatment (Figure 3 B,C). We selected these two major hits for in‐depth validation. U2SURP is a spliceosomal factor and NOP14 (which showed the highest shift) is crucial for 40S ribosome subunit formation as well as maturation of 18S rRNA.^13^

**Figure 3:**
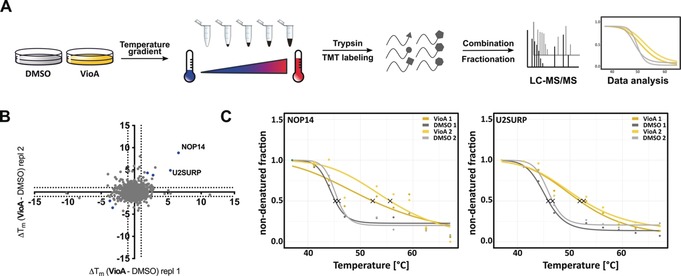
Target identification of VioA in Jurkat cells using thermal proteome profiling. A) Schematic workflow of the thermal proteome profiling experiment. Living Jurkat cells were treated with 1 μm VioA or DMSO for 1 hour. Subsequently, aliquots were incubated at 9 different temperatures (37 °C–67 °C), centrifuged, and the proteins digested in supernatant. After TMT labeling and HILIC fractionation, the samples were analyzed by mass spectrometry. B) Scatterplot of *T*_m_ shifts determined from two biological replicates of VioA vs. DMSO treatment. Melting‐point shifts passing all significance criteria (see Supporting Information) are shown in blue. Data was preprocessed using MaxQuant^14^ and thermal‐response curve fitting as well as melting‐point calculations were carried out using the TPP R package.^9^ C) Thermal‐response curves and calculated melting points (asterisks) for NOP14 and U2SURP proteins of VioA‐treated (orange) and DMSO‐treated (grey) cells. Two biological replicates are shown.

U2SURP was excluded as a potential target of VioA because splicing was not affected in VioA‐treated cells, as shown by dual luciferase splicing reporter assay (Figure S8). NOP14 plays a crucial role in the initial steps of ribosome biogenesis, namely, the formation of the small subunit processome (SSU processome), an early stage of the formation of the small ribosomal subunit (40S). This processing is enhanced in cancer cells due to the high demand for continuous ribosome production for their extensive growth.^15^, ^16^ In eukaryotes, ribosome biogenesis takes place in the nucleoli that contain genes encoding for ribosomal RNAs (rRNAs), which build the nucleic acid backbone of the ribosome.^15^ Interestingly, NOP14 siRNA knockdown cells (knockdown confirmation shown in Figure S9) as well as VioA‐treated cells display reduced rRNA levels in nucleoli, as demonstrated by 5‐FU immunostaining (Figure 4 A,B). Actinomycin D (ActD), which inhibits Pol I transcription, served as positive control. rDNA transcription is necessary for the formation of nucleoli, whereas perturbations of the transcriptional machinery leads to their disassembly.^15^ Consistently, ActD treatment led to displacement of NOP14 in the whole nucleus due to nucleolar disruption, whereas VioA treatment did not alter the localization of NOP14. Based on these results, VioA obviously does not interfere with ribosome biogenesis at the level of transcription (Figure 4 C). Importantly, knockdown of NOP14 impairs cancer‐cell proliferation (Figure 4 D) as shown before for VioA (Figure 1 B, Figure 2 A).

**Figure 4:**
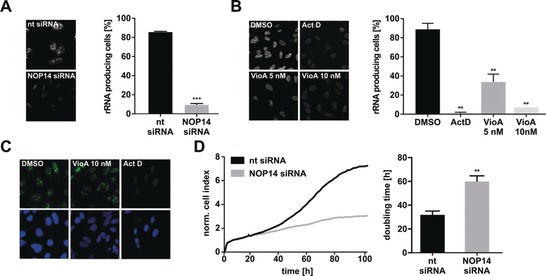
Target verification. A) Nuclear run‐on assay with HeLa cells transfected with non‐targetted (nt) or NOP14 siRNA for 48 h. Cells with nucleolar rRNA foci were counted as rRNA‐producing cells. Bars show the mean±SEM of three independent experiments performed in duplicate, two‐tailed unpaired Student's t‐test, *** *P*<0.001. B) Nuclear run‐on assay with HeLa cells treated with VioA for 24 h or treated with 6 μm actinomycin D (ActD) for 2 h as a positive control. Counting of positive cells (lower panel) same as in (A). Bars (upper panel) represent the mean±SEM of three independent experiments performed in duplicate, one‐way ANOVA, Dunnett's test, ** *P*<0.002. C) HeLa cells were treated with 10 nm VioA or 6 μm ActD for 2 h and immunostained for NOP14. Nuclei were stained with Hoechst. Representative images out of three independent experiments are shown. D) xCELLigence real‐time proliferation measurement of HeLa cells either transfected with nt or NOP14 siRNA for 24 h before seeding into E‐plates. Proliferation was monitored for the subsequent 72 h. Representative curve out of three independent experiments is shown. Respective doubling time values were calculated using xCELLigence RTCA software. Bars represent the mean±SEM of three independent experiments performed in triplicate, two‐tailed unpaired Student's t‐test, ** *P*<0.002.

Various protein–protein interactions with NOP14 are essential for a functional ribosome biogenesis machinery in yeast.^13^, ^17^ Thus, we focused on unravelling the interactome of NOP14 through MS‐based co‐immunoprecipitation (co‐IP) experiments. Co‐IP (with an immobilized anti‐NOP14 antibody) was carried out in presence of a DSSO crosslinker to enable the trapping of transient binding partners as applied previously (Figure 5 A).^18^ Analysis of pulled‐down proteins by LC–MS/MS identified several significantly enriched proteins (Table S3) compared to the isotype control co‐IP (Figure 5 B) in Jurkat cells, amongst others, nucleolar complex protein 4 homolog (NOC4L) and ribosomal RNA small subunit methyltransferase NEP1 (EMG1). In order to identify VioA‐sensitive interactions of NOP14, the co‐IP experiment was repeated with compound treatment for 24 hours prior to in situ DSSO crosslinking (Figure S10, Table S4). Treatment with VioA led to the depletion of various proteins enriched in non‐treated co‐IP experiments (Table S4). Since it is known that interaction of NOP14 with both NOC4L and EMG1 is essential for proper maturation of 18S rRNA and 40S assembly and export,^13^, ^19^ we investigated the influence of VioA treatment on the interplay of those proteins.

**Figure 5:**
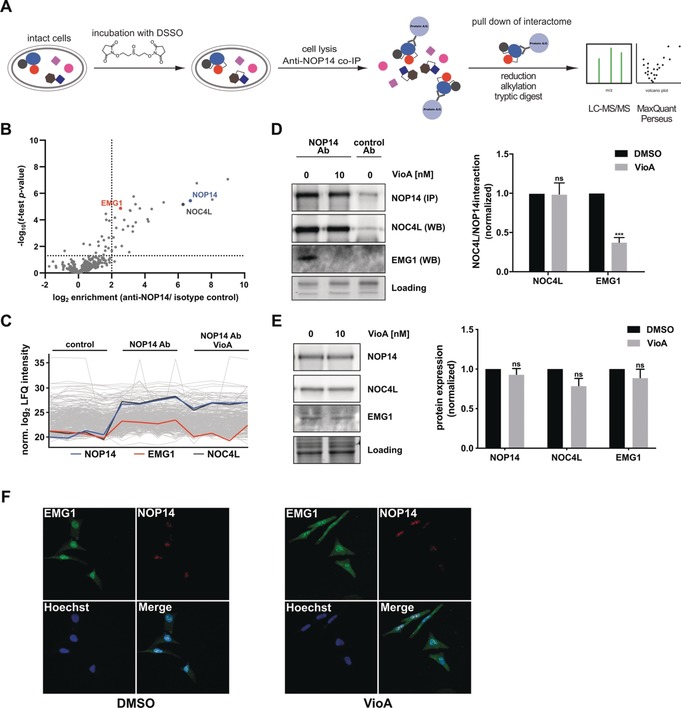
Co‐immunoprecipitation with anti‐NOP14 antibody. A) Schematic workflow of MS‐based co‐immunoprecipitation. Living cells were treated with 10 nm **VioA** or DMSO, respectively, for 24 hours. Subsequently, DSSO linker was added to covalently link protein complexes within cells. NOP14 and linked proteins were enriched using an anti‐NOP14 antibody immobilized on protein A/G beads. Tryptic peptides were measured via LC‐MS/MS, and analyzed using MaxQuant^14^ and Perseus.^21^ B) Volcano plot represents two‐sample t‐test results of anti‐NOP14 co‐IP compared to isotype control co‐IP (*n*=4). Cutoff criteria were defined as log_2_=2 (4‐fold enrichment) enrichment factor and ‐log_10_ (t‐test *p*‐value)=1.3 (dotted lines). NOP14 is colored in blue, NOC4L in dark grey and EMG1 in orange. C) Comparison of normalized LFQ intensities of NOP14, NOC4L and EMG1 in isotype control and anti‐NOP14 co‐IP samples treated with 10 nm VioA prior to co‐IP after missing value imputation. D) Co‐IP of NOP14 in Jurkat cells and detection of NOP14 and interactors NOC4L/ EMG1 by western blot analysis. A representative experiment out of three independent experiments is shown. Bars represent mean±SEM of three independent experiments, two‐tailed unpaired Student's t test, *** *P*<0.001 E) Western blot analysis of NOP14, NOC4L and EMG1 expression levels of Jurkat cells either treated with DMSO or 10 nm VioA for 24 h. Bars represent the mean±SEM of three independent experiments, two‐tailed unpaired Student's t test, ns *P*>0.05. F) HeLa cells treated with VioA were co‐stained for NOP14 (red) and EMG1 (green). Nuclei were stained with Hoechst 33342. Representative images out of three independent experiments performed in duplicate are shown.

Remarkably, MS‐based and western blot based co‐IP experiments revealed that VioA treatment led to a selective loss of interaction between NOP14 and EMG1, whereas the NOP14–NOC4L association remained unperturbed (Figure 5 C,D). Of note, expression levels of neither NOC4L nor EMG1 were affected by VioA (Figure 5 E).

In yeast, EMG1 is involved in the modification of uridine 1191 of the 18S rRNA. Its catalytic activity is not essential for ribosome biogenesis, however the presence of the protein within the 90S pre‐ribosome is inevitably required.^20^ Nucleolar localization of NOP14 depends on binding to NOC4L.^22^ This interaction is not disturbed by VioA, hence, NOP14 remains in the nucleolus upon VioA treatment. However, since the physical interaction of NOP14 with EMG1 is required for nuclear localization of EMG1,^13^ impaired binding of those two poteins upon VioA treatment led to a distribution of EMG1 in the entire cell in comparison to control cells, where EMG1 is mainly located in the nucleus (Figure 5 F).

In conclusion, vioprolides represent a unique class of natural products with potent bioactivity towards cancer cells in the nanomolar range. Using TPP, we identified NOP14, which is involved in ribosome biogenesis, as an unprecedented interaction partner of VioA. NOP14 plays a key role in the assembly of the small ribosomal subunit and in fact, VioA is the first natural product reported that addresses this crucial pathway. PBMCs showed low response to VioA in contrast to ALL cancer cells, thus suggesting VioA as a promising lead structure for cancer‐cell targeting. Closer mechanistic studies showed that VioA alters the interaction of NOP14 with EMG1, leading to delocalization of EMG1. Our results indicate that targeting the NOP14‐EMG1 interaction, and thereby ribosome biogenesis, is a promising anticancer strategy and underline the great potential of VioA as a chemical tool to study ribosomal processes as well as starting point for future drug development.

The MS data have been deposited to the ProteomeXchange Consortiom via the PRIDE^23^ partner repository with the dataset identifier PXD015196.

## Conflict of interest

The authors declare no conflict of interest.

## Supporting information

As a service to our authors and readers, this journal provides supporting information supplied by the authors. Such materials are peer reviewed and may be re‐organized for online delivery, but are not copy‐edited or typeset. Technical support issues arising from supporting information (other than missing files) should be addressed to the authors.

Supplementary
