# Supplementary material for: The Cytotoxic Natural Product Vioprolide A Targets Nucleolar Protein 14, Which Is Essential for Ribosome Biogenesis
Source: Angew Chem Int Ed Engl. 2019 Dec 12;59(4):1595–600. doi: 10.1002/anie.201911158 (PMC7004033; doi:10.1002/anie.201911158)
Supplement: Supplementary file 1 — Supplementary [file ANIE-59-1595-s001.pdf]

Angewandte Chemie
Eine Zeitschrift der Gesellschaft Deutscher Chemiker

Supporting Information

The Cytotoxic Natural Product Vioprolide A Targets Nucleolar Protein 14, which is Essential for Ribosome Biogenesis

Volker C. Kirsch+, Christina Orgler+, Simone Braig, Irmela Jeremias, David Auerbach, Rolf Müller, Angelika M. Vollmar, and Stephan A. Sieber**

anie_201911158_sm_miscellaneous_information.pdf

1. SUPPLEMENTARY FIGURES 3

2. SUPPLEMENTARY TABLES 14

3. SUPPLEMENTARY SCHEMES 18

4. BIOCHEMICAL PROCEDURES 19

5. PROTEOMICS METHODS 23

6. TARGETED METABOLIC ASSAY 31

7. BIOINFORMATICS 33

8. CHEMICAL SYNTHESIS 36

9. NMR AND LC-MS SPECTRA 39

10. GLOSSARY 42

11. SUPPLEMENTARY REFERENCES 44

Important Note

The mass spectrometry proteomics data have been deposited to the ProteomeXchange Consortium via the PRIDE[1] partner repository with the data identifier PXD015196.

1. Supplementary figures

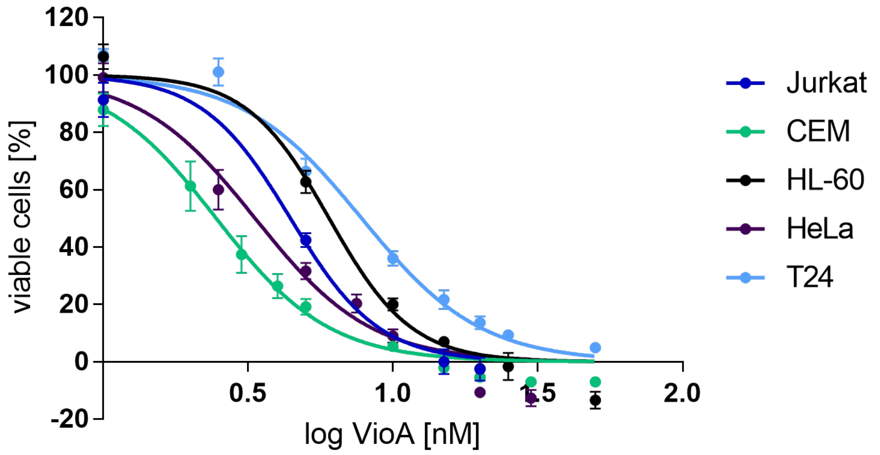

Figure S1: Inhibition of proliferation of different cell lines treated with vioprolide A for 72h. Number of viable cells was determined by cell titer blue assay (Jurkat, CEM, and HL-60) or crystal violet assay (HeLa, T24) and normalized towards DMSO control. Data points represent the mean ± SEM of three independent experiments performed in triplicate.

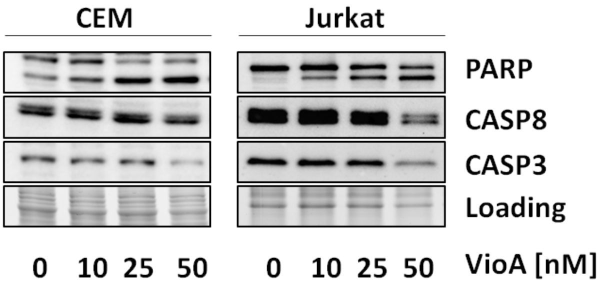

Figure S2: Confirmation of VioA induced apoptotic cell death by western blot analysis of caspase activation and PARP cleavage. Jurkat and CEM cells were treated with **VioA** for 24h and protein levels of procaspase-3 (CASP3), procaspase-8 (CASP8) and cleavage of poly ADP ribose polymerase (PARP) were determined. Representative experiments out of three biological replicates are shown.

Of a wide range of stimuli and conditions that can trigger apoptosis in cells, there are two main pathways to distinguish: the intrinsic (mitochondria-mediated) and the extrinsic (death receptor mediated) pathway. To decipher, which pathway is addressed by **VioA** treatment, characteristics of mitochondria-mediated apoptosis were examined in Jurkat cells. Mitochondrial membrane permeabilization is considered a general attribute of mitochondria-mediated cell death and represented as a “point of no return” event that results in apoptosis. Under physiological conditions, mitochondria display a high mitochondrial membrane potential ($\Delta\Psi m$) while in cells undergoing apoptosis $\Delta\Psi m$ is dissipating[2]. Thereby, the pro-apoptotic Bcl-2 family members BAX and BAK are recruited to the outer mitochondrial membrane (OMM) and oligomerize to mediate mitochondrial outer membrane permeabilization (MOMP), resulting in cytochrome C release and caspase activation [3].

Here, we used the lipophilic cationic molecular probe JC-1 to measure the alterations of $\Delta\Psi m$ in **VioA** treated cells as the dye exhibits $\Delta\Psi m$ dependent aggregation. Of note, loss of $\Delta\Psi m$ could be demonstrated by decreased red/ green fluorescence ratio in **VioA** treated Jurkat cells after 24h (Figure S3A). Moreover, antiapoptotic Bcl-2 family members play crucial roles in the mitochondrial apoptotic pathway by regulating MOMP [4]. VioA strikingly downregulated the expression levels of antiapoptotic Bcl-2 family proteins Bcl-2, Bcl-xL and Mcl-1 after 24h treatment (Figure S3B). To further test the role of anti-apoptotic Bcl-2 family members on **VioA**- induced apoptosis, Bcl-2 and Bcl-xL overexpressing Jurkat cells were challenged with **VioA**. Additionally, a Jurkat cell line deficient for caspase-8 was tested (Figure S3E). Caspase-8 plays a role in the extrinsic apoptotic pathway execution that is, independently from the intrinsic pathway, activated upon death ligand (e.g. FasL) binding to death receptors and receptor ligation.[5] Jurkat cells overexpressing Bcl-2 or Bcl-xL were significantly less sensitive towards **VioA** treatment than the empty vector control cell line Jurkat neo, whereas Jurkat Casp8 -/- exposed a sensitivity towards **VioA** equally to Jurkat neo (Figure S3C-D). These

results indicate that apoptosis induction by **VioA** can only be effectively executed upon functional mitochondrial apoptosis signalling and that the extrinsic pathway is not pivotal for **VioA** induced apoptosis.

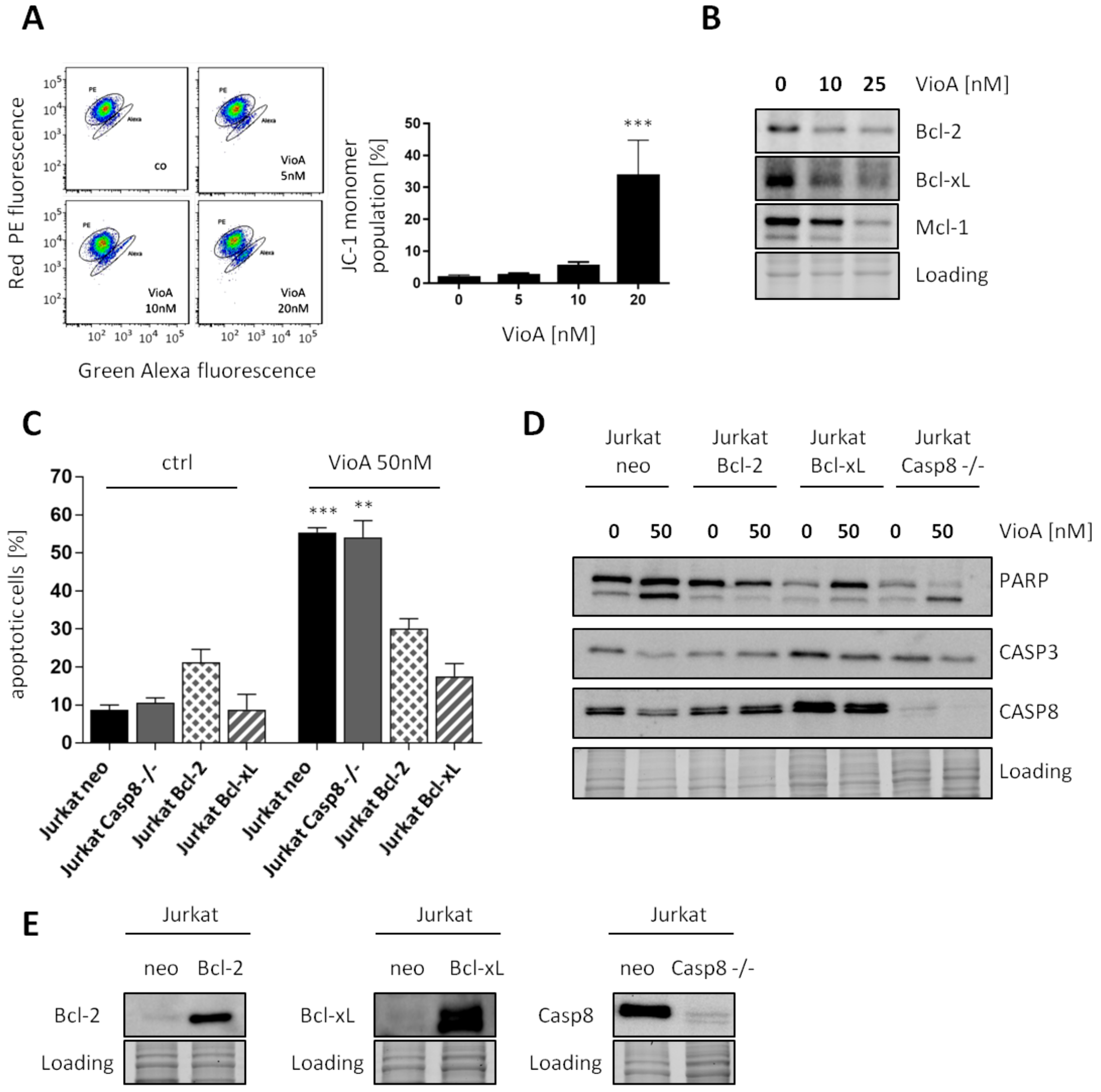

Figure S3: Vioprolide A causes mitochondria-mediated apoptosis. (**A**) JC-1 was used as fluorescent probe for cytometric estimation of mitochondrial membrane potential (Δϕm) in Jurkat cells treated with vioprolide A (**VioA**) as indicated for 24h. The green fluorescent JC-1 monomer population represents cells with loss of Δϕm and was quantitatively measured by FACS analysis, one-way ANOVA, Dunnett's test, *** P<0.001. (**B**) Expression levels of the anti-apoptotic mitochondrial proteins Bcl-2, Bcl-xL and Mcl-1 decline upon **VioA** treatment as indicated for 24h. (**C**) Apoptosis induction is suppressed in Bcl-2 and Bcl-xL overexpressing Jurkat cells respectively (Jurkat Bcl-2, Jurkat-Bcl-xL) compared to the empty vector control cell line (Jurkat neo). Caspase-8 deficiency (CASP8 -/-) does not rescue Jurkat cells from apoptosis induction by **VioA.** Percentage of apoptotic cells was determined by propidium iodide staining and flow cytometry after 48 h, two-tailed, unpaired Student's t-test, ** P<0.002, *** P<0.001. (**D**) Induction of apoptosis analyzed by immunoblotting. Jurkat cells were treated with **VioA** as indicated for 24h and protein levels of caspase-3 (CASP3), caspase-8 (CASP8) and poly ADP ribose polymerase (PARP) were determined. (**E**) Verification of Bcl-2 and Bcl-xL overexpression as well as caspase-8 deficiency by Western Blot. (A, C) All bars represent the mean ± SEM of three independent experiments performed in triplicates. (B, D, E) Representative experiments out of three independent experiments are shown.

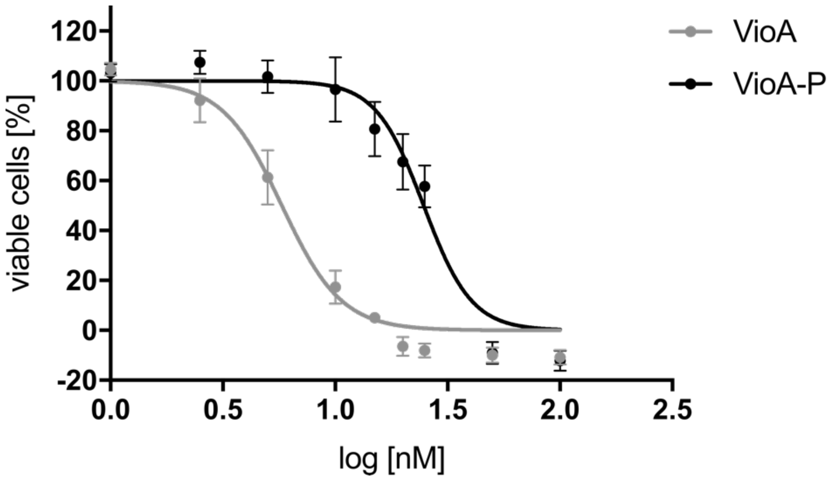

Compound	IC_{50} [nM]
VioA	5.8 ± 0.4
VioA-P	24.7 ± 1.7

Figure S4: Inhibition of proliferation of Jurkat cancer cells treated with VioA or VioA-P for 72h. Number of viable cells and corresponding IC_{50} values were determined by cell titer blue assay and normalized towards DMSO control. Data points represent the mean ± SEM of three independent experiments performed in triplicates.

A

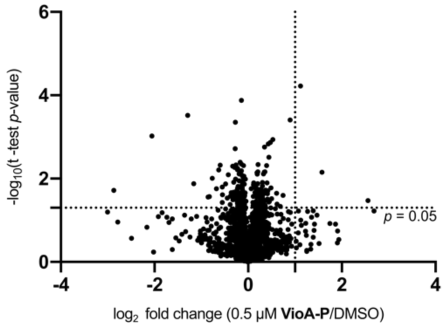

log_2 enrichment	$-log_{10}$ (*t*-test *p*-value)	Gene	Protein
2.56	1.47	C14orf1	Probable ergosterol biosynthetic protein 2B
1.58	2.15	NACC1	Nucleus accumbens-associated protein 1
1.12	4.22	ZMPSTE24	CAAX prenyl protease 1 homolog

B

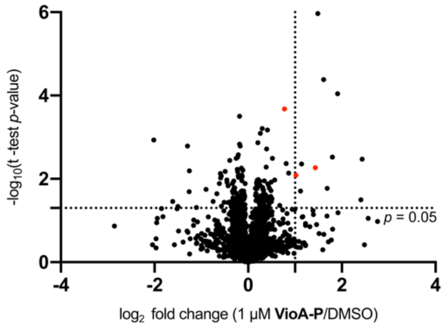

log_2 enrichment	$-log_{10}$ (*t*-test *p*-value)	Gene	Protein
2.43	2.47	SET	Protein SET
2.41	1.50	C14orf1	Probable ergosterol biosynthetic protein 2B
1.91	4.04	SLC12A7	Solute carrier family 12 member 7
1.80	2.52	CALU	Calumenin
1.68	1.77	PLD3	Phospholipase D3
1.61	4.38	ZMPSTE24	CAAX prenyl protease 1 homolog
1.48	5.97	CD3E	T-cell surface glycoprotein CD3 epsilon chain
1.43	2.27	CSTD	Cathepsin D
1.14	2.36	COMT	Catechol O-methyltransferase
1.12	1.71	RAD23A/B	UV excision repair protein RAD23 homolog A/B
1.02	2.09	HMXO2	Heme oxygenase 2
0.78	3.68	VDAC1	Voltage-dependent anion-selective channel protein 1

C

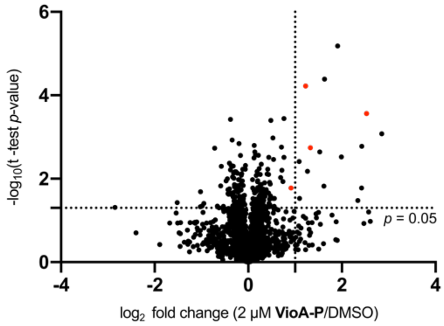

log_2 enrichment	$-log_{10}$ (*t*-test *p*-value)	Gene	Protein
2.52	3.56	CTSD	Cathepsin D
2.42	2.78	SET	Protein SET
2.42	1.78	GOPC	Golgi-associated PDZ and coild-coil motif containing protein
2.34	1.48	SLC25A20	Mitochondrial carnitine/acylcarnitine carrier protein
1.99	2.52	PLD3	Phospholipase D3
1.91	5.18	CD3E	T-cell surface glycoprotein CD3 epsilon chain
1.63	4.39	ZMPSTE24	CAAX prenyl protease 1 homolog
1.62	1.82	TIMM17B	Mitochondrial import inner membrane translocase subunit TIM17-B
1.53	2.64	CALU	Calumenin
1.33	2.74	SCCPDH	Saccharopine dehydrogenase-like oxidoreductase
1.26	2.18	DNAJB6	DnaJ homolog subfamily B member 6
1.23	4.22	VDAC1	Voltage-dependent anion-selective channel protein 1
1.10	1.53	CYB5B	Cytochrome b5 type B
1.09	2.41	HM13	Minor histocompatibility antigen H13
0.92	1.78	HMXO2	Heme oxygenase 2

Figure S5: Quantitative proteome enrichment analysis of VioA-P treated Jurkat cells. Volcano plots of *in situ* label-free AfBPP experiments with (**A**) 0.5 µM, (**B**) 1.0 µM and (**C**) 2.0 µM **VioA-P** treated intact Jurkat cells compared to DMSO treated cells (n=3). Proteins significantly enriched (log_2 enrichment > 1, $-log_{10}$ (*t*-test *p*-value) > 1.3) are listed in respective tables. Additionally, known diazirine background binders (as detected in A549 and HeLa cells)[6] are indicated as red dots, and marked in red in tables.

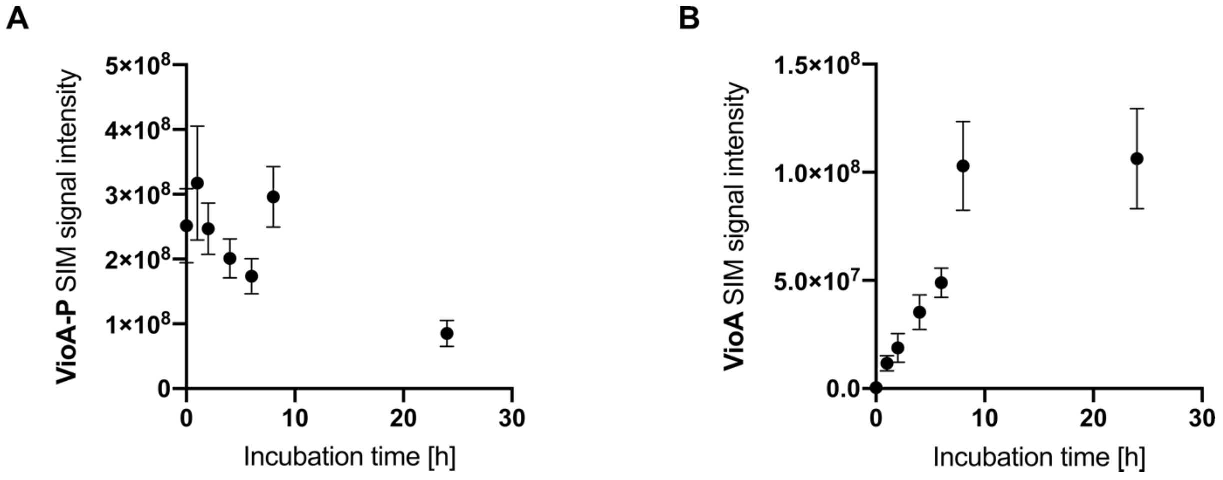

Figure S6: Time dependent hydrolysis of VioA-P in Jurkat cell lysate as detected by targeted metabolic degradation assay. VioA-P was incubated with Jurkat cell lysate, an aliquot sampled at indicated time points and metabolic reactions quenched by the addition of cold MeCN. Subsequently, samples were analyzed via LC-MS in SIM mode targeting molecules of interest. Analysis revealed a time-dependent decrease of **VioA-P** (**A**) observed with a concurrent time-dependent increase of **VioA** signal intensity (**B**), indicating that **VioA-P** is hydrolyzed over time to **VioA**. Data points represent the mean ± SEM of three independent experiments.

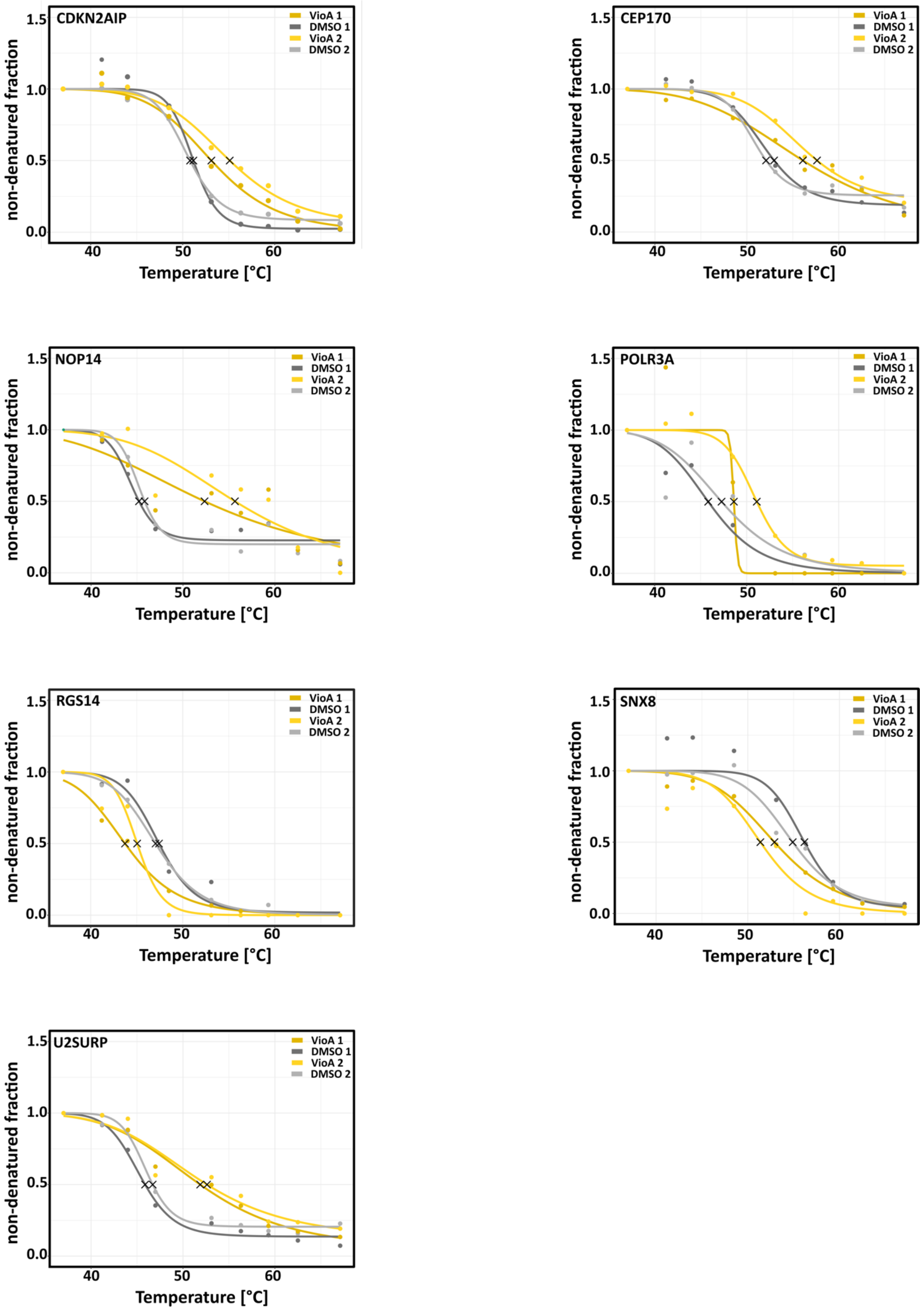

Figure S7: **Thermal melting curves of potential hits identified in thermal proteome profiling experiment.** Thermal response curves for proteins passing all filtering thresholds (see data analysis for TPP experiments) when treating cells with 1 µM **VioA** (orange) compared to DMSO controls (grey). Melting points (asterisks) and melting curve fitting was carried out and results visualized using the TPP

R package[7] and Graphpad Prism 8. CEP170 met all significance criteria in one replicate but was just outside the threshold for significance in the other.

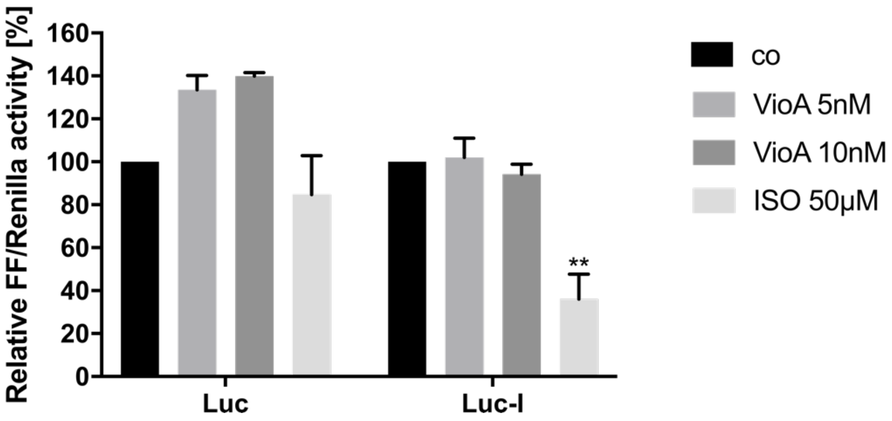

Figure S8: Splicing Dual Luciferase Reportergene Assay Relative expression of the luciferase reporters in HeLa cells. HeLa cells were transfected either with Luc or Luc-I firefly luciferase reporters and co-transfected with renilla luciferase control reporter for 24h with FUGENE HD reagent according to manufacturer's protocol. After transfection, cells were treated with VioA or the pre-mRNA splicing inhibitor Isoginkgetin (ISO) for 24h. Luciferase activity was measured using Dual Luciferase Assay Kit (Promega) according to manufacturer's protocol. Relative firefly/renilla luciferase activity was calculated and values were normalized towards untreated control. Bars represent the mean ± SEM of three independent experiments performed in duplicate, one-way ANOVA, Dunnett's test, ** P<0.002.

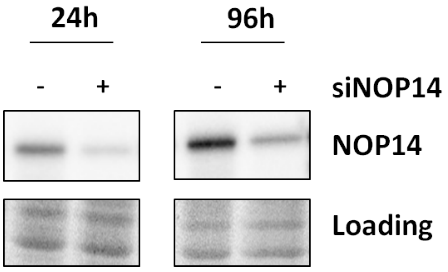

Figure S9: Verification of NOP14 siRNA knockdown in HeLa cells. HeLa cells were either transfected with non-targeted siRNA (-) or siRNA specific for NOP14 (+) for the indicated time points before cell lysis and western blot analysis with an anti-NOP14 antibody. Representative blots are shown.

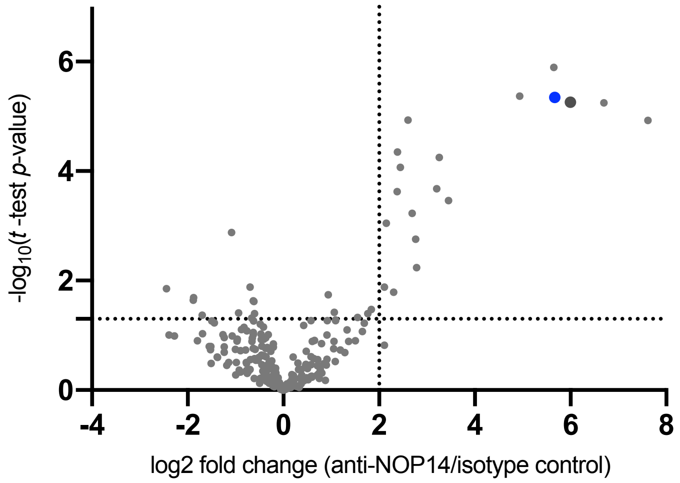

Figure S10: Volcano plot of co-IP of NOP14 against isotype control in Jurkat cells treated with VioA prior to co-IP enrichment. Cells were treated with VioA (10 nM) for 24h prior to incubation with 2 mM DSSO crosslinker for chemical cross-linking of protein-protein interactions and subsequent pull down using an anti-NOP14 antibody or isotype control. Volcano plot represents t test results of anti-NOP14 co-IP compared to isotype control co-IP (n=4 for each experiment). Cutoff criteria were defined as $\log_2$ enrichment > 2 and $-\log_{10}$ (*t*-test *p*-value) > 1.3 (dotted lines). NOP14 (blue) and NOC4L (dark grey) are highlighted in the plot. Significant enriched proteins are listed in Table S4. EMG1 is not present in the plot as it is only detected in one of four anti-NOP14 antibody pulldown samples.

2. Supplementary tables

Table S1: Clinical characteristics of patients from which PDX cells were derived are shown.

Sample	Disease stage	Age	Sex	Subtype	Karyotype
ALL-50	initial diagnosis	7	female	pre-B	t(1;19)
ALL-199	relapse 2	8	female	pre-B	somatic trisomy 21
ALL-707	initial diagnosis	2	male	pro-B	t(4;11)

Table S2: Calculated *p*-value, slope and curve fit for protein targets of vioprolide A in Jurkat cells as elucidated by *in situ* thermal proteome profiling. Proteins listed met all filter criteria.[7] CEP170 met all significance criteria in one replicate but was just outside the threshold for significance in the other. Cutoff criteria for: p-value < 0.05 in one replicate, < 0.1 in the second replicate. R^2 fit > 0.8, min. slope < -0.06.

T_m shift repl 1/2 [°C]	p-value adj. repl 1/2	R^2 curve fit (VioA1/DMSO1/VioA2/DMSO2)	Min. slope VioA vs DMSO repl. 1/2	Gene	Protein
6.61/8.85	6.5E-07/9.32E-14	0.81/0.94/0.84/0.95	-0.10/-0.10	NOP14	Nucleolar protein 14
5.39/4.89	5.18E-03/2.91E-02	0.99/0.99/0.95/0.99	-0.08/-0.09	U2SURP	U2 snRNP-associated SURP motif-containing protein
3.13/5.55	3.13E-01/4.00E-05	0.98/0.99/0.99/0.99	-0.09/-0.11	CEP170	Centrosomal protein of 170 kDa
1.96/4.31	4.93E-02/3.48E-03	0.99/0.98/0.99/1.00	-0.18/-0.13	CDKN2AIP	CDKN2A-interacting protein
2.79/3.82	6.63E-03/1.2E-03	0.82/0.96/0.99/0.84	-1.29/-0.14	POLR3A	DNA-directed RNA polymerase subunit
-3.70/-2.00	3.32E-02/1.55E-01	0.99/0.98/0.96/1.00	-0.13/-0.19	RGS14	Regulator of G-protein signaling 14
-3.27/-3.53	8.03E-02/8.61E-02	0.99/0.93/0.92/0.98	-0.12/-0.09	SNX8	Sorting nexin-8

Table S3: Proteins enriched in MS-based co-IP experiments.
Cells were treated with DMSO for 24h prior to incubation with 2 mM DSSO crosslinker for chemical cross-linking of protein-protein interactions and subsequent pull down using an anti-NOP14 antibody or isotype control (n=4). Significantly enriched proteins (log_2 enrichment > 2, -log_{10} *t*-test *p*-value > 1.3) are listed.

log_2 enrichment	-log_{10} (*t*-test *p*-value)	Gene	Protein
8.99	7.06	TFG; TFG/ALK fusion	Protein TFG; Tyrosine-protein kinase receptor
8.06	5.53	SNAP29	Synaptosomal-associated protein 29
7.12	6.76	NANS	Sialic acid synthase
6.75	5.46	NOP14	Nucleolar protein 14
6.30	5.18	NOC4L	Nucleolar complex protein 4 homolog
5.13	5.32	DECR	2,4-dienoyl-CoA reductase, mitochondrial
4.41	4.82	MTCL1	Microtubule cross-linking factor 1
4.21	3.88	DTWD2	DTW domain-containing protein 2
4.16	4.05	NACC1	Nucleus accumbens-associated protein 1
3.70	4.86	IPO5	Importin-5
3.45	3.82	SEPT6	Septin 6
3.44	5.18	SEPT7	Septin 7
3.32	5.76	TPR	Nucleoprotein TPR
3.27	3.51	CIZ1	Cip1-interacting zinc finger protein
3.27	3.11	GFPT1	Glutamine-fructose-6-phosphate aminotransferase 1
3.12	4.68	PRKCA	Protein kinase C alpha type
3.10	4.72	SEPT2	Septin 2
3.06	1.57	SUB1	Activated RNA polymerase II transcriptional coactivator p15
2.98	3.53	HNRNPA2B1;HNRPA2B1	Heterogeneous nuclear ribonucleoproteins A2/B1
2.75	4.00	NAPA	Alpha-soluble NSF attachment protein
2.69	3.27	IST1	IST1 homolog
2.61	2.03	PPP2R1A	Serine/threonine-protein phosphatase 2A 65 kDa regulatory subunit A alpha isoform
2.48	4.89	EMG1	Ribosomal RNA small subunit methyltransferase NEP1
2.28	3.39	HSD17B10	3-hydroxyacyl-CoA dehydrogenase type-2
2.24	1.95	PPA1	Inorganic pyrophosphatase
2.20	4.17	ATAD3A/B	ATPase family AAA domain-containing protein 3A/B
2.11	2.68	EEF1D	Elongation factor 1-delta
2.01	1.66	PCBP2	Poly(rC)-binding protein 2

Table S4: Proteins enriched in MS-based Co-IP experiments after treatment with VioA for 24h. Cells were treated with **VioA** (10 nM) or DMSO, respectively prior to incubation with 2 mM DSSO crosslinker for chemical cross-linking of protein-protein interactions and subsequent pull down using an anti-NOP14 antibody or isotype control (n=4). Significantly enriched proteins ($\log_2$ enrichment > 2, $-\log_{10}$ *t*-test *p*-value > 1.3) are listed.

$\log_2$ enrichment	$-\log_{10}$ (*t*-test *p*-value)	Gene	Protein
7.61	4.93	TFG; TFG/ALK fusion	Protein TFG; Tyrosine-protein kinase receptor
6.70	5.24	SNAP29	Synaptosomal-associated protein 29
6.00	5.26	NOC4L	Nucleolar complex protein 4 homolog
5.67	5.35	NOP14	Nucleolar protein 14
5.65	5.90	NANS	Sialic acid synthase
4.94	5.37	DECR	2,4-dienoyl-CoA reductase, mitochondrial
3.45	3.46	MTCL1	Microtubule cross-linking factor 1
3.26	4.25	GFPT1	Glutamine-fructose-6-phsphate aminotransferase [isomerizing] 1
3.21	3.68	DTWD2	DTW domain-containing protein 2
2.78	2.24	IPO5	Importin-5
2.76	2.75	CIZ1	Cip1-interacting zinc finger protein
2.69	3.23	TPR	Nucleoprotein TPR
2.60	4.93	SEPT7	Septin-7
2.44	4.07	HNRNPA2B1;HNRPA2B1	Heterogeneous nuclear ribonucleoproteins A2/B1
2.39	4.35	NAPA	Alpha-soluble NSF attachment protein
2.38	3.63	HSD17B10	3-hydroxyacyl-CoA dehydrogenase type-2
2.30	1.79	SEPT2	Septin-2
2.15	3.05	PPA1	Inorganic pyrophosphatase
2.11	1.88	SEC22B	Vesicle-trafficking protein SEC22b

Table S5: Reporter ion isotopic distributions as stated in product data sheet for TMT10plex^{TM} Label reagent set (LOT number:SF239894). Reporter ion isotopic distributions were used as isotope correction factors in MaxQuant data analysis.

Mass Tag	Reporter Ion	-2	-1	Monoisotopic	+1	+2
TMT^{10}-126	126.127726	0.0%	0.0%	100%	6.9%(127C)	0.1%(128C)
TMT^{10}-127N	127.124761	0.0%	0.4%	100%	7.3%(128N)	0.2%(129N)
TMT^{10}-127C	127.131081	0.0%	0.6%(126)	100%	5.9%(128C)	0.0%(129C)
TMT^{10}-128N	128.128116	0.0%	0.4%(127N)	100%	4.1%(129N)	0.0%(130N)
TMT^{10}-128C	128.134436	0.0%(126)	1.4%(127C)	100%	5.1%(129C)	0.0%(130C)
TMT^{10}-129N	129.131471	0.0%(127N)	1.4%(128N)	100%	5.0%(130N)	0.0(131)
TMT^{10}-129C	129.137790	0.0%(127C)	2.3%(128C)	100%	4.3%(130C)	0.0%
TMT^{10}-130N	130.134825	0.0%(128N)	2.7%(129N)	100%	3.9%(131)	0.0%
TMT^{10}-130C	130.141145	0.0%(128C)	2.9%(129C)	100%	3.3%	0.0%
TMT^{10}-131	131.138180	0.0%(129N)	3.4%(130N)	100%	3.3%	0.0%

Table S6: Overview of TMT slots used for incubation with temperature dependent sample fractions.

Mass Tag	Temperature point
TMT^{10}-126	37.0°C
TMT^{10}-127N	41.2°C
TMT^{10}-127C	44.0°C
TMT^{10}-128N	48.5°C
TMT^{10}-129N	53.1°C
TMT^{10}-129C	56.3°C
TMT^{10}-130N	59.3°C
TMT^{10}-130C	62.5°C
TMT^{10}-131	67.1°C

3. Supplementary schemes

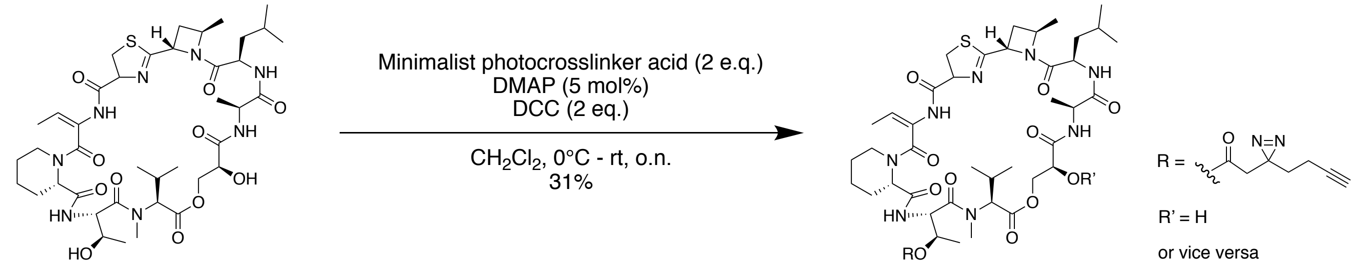

Scheme S1: Synthesis of **VioA-P**. Two possible esters can be formed upon coupling with the photocrosslinker acid (R vs. R´). A single HPLC peak suggests selective product formation (Scheme S2). However, the exact location could not be determined by analytical methods including MS-fragmentation and NMR (see section 9).

4. Biochemical procedures

Cell lines

Jurkat cells (wild-type, CASP8- deficient, Bcl-2- and Bcl-xL- overexpressing) were kindly provided by P.H. Krammer (Heidelberg, Germany) and cultured with RPMI 1640 containing FCS 10%, pyruvate 1mM and penicillin/streptomycin (P/S). CCRF-CEM cells were obtained from M. Kavallaris (Sydney, Australia) and maintained with RPMI 1640 containing FCS 10% and P/S. HeLa and T24 cells were provided by the DSMZ (Braunschweig, Germany) and cultured in DMEM containing FCS 10% and P/S. HL-60 cells from ATCC (Manassas, VA, USA) were maintained in IMDM medium supplemented with FCS 20% and P/S. All cells were cultured at 37°C with 5% CO_2 at constant humidity.

Patient-derived xenograft cells and peripheral blood mononuclear cells

The preclinical mouse model for acute leukemia using patients' cells has already been described previously.[8] PDX cells were freshly isolated from the bone marrow or spleen of NOD.Cg-*Prkdc*^{scid} *Il2rg*^{tm1Wjl}/SzJ (NSG) mice and cultured in RPMI 1640 containing FCS 20%, pyruvate 1mM and P/S. Patients clinical characteristics are summarized in table S1. Written informed consent was obtained from all patients. The study was performed in accordance with the ethical standards of the responsible committee on human experimentation (written approval by the Research Ethics Boards of the medical faculty of Ludwig-Maximilians-University, Munich, numbers 068-08 and 222-10) and with the Helsinki Declaration of 1975, as revised in 2000. Peripheral blood mononuclear cells were freshly isolated from EDTA- anticoagulated blood of healthy donors by gradient centrifugation using Ficoll-Paque PLUS (GE Healthcare, Chicago, IL, USA) according to manufacturer's protocol. The cells were maintained in RPMI 1640 with 2mM glutamine and 1mM pyruvate and supplemented with FCS 20%.

Transfection of cells

Silencing of NOP14 was performed with DharmaFECT I Transfection reagent (Thermo Scientific, Waltham, MA, USA) corresponding to the manufacturer's recommendation. The following siRNAs were used: non-targeting (nt) siRNA (D-001810-01) and ON-TARGETplus human NOP14 siRNA – SMARTpool (Dharmacon, Lafayette, CO, USA).

Cell viability

Cells were seeded into 96- well plates and incubated for 3h (suspension cells) or 24h (adherent cells). Cells were treated as indicated for further 72h. Cell viability of suspension cells was assessed by CellTiter- Blue (Promega, Madison, WI, USA) assay according to manufacturer's instructions. For adherent cell lines, crystal violet staining was used to determine the number of viable cells.

xCELLigence assay

For real-time monitoring of HeLa cell proliferation the RTCA xCELLigence device was used. Briefly, transfected cells were seeded in equilibrated E-plates 16 and after attachment, their response was continuously monitored for the subsequent 72h at 37°C. Through impedance measurement, the xCELLigence system evaluates the cell index, a dimensionless parameter, which is proportional to the cell number. The cell index was normalized at the time-point of treatment and The RTCA software was used for further data analysis.

Apoptosis and cell cycle analysis

Apoptosis rate was determined by propidium iodide (PI) staining and flow cytometry as described by Nicoletti et al.[9] Cells were seeded into 24 well plates and treated with test compounds as indicated. Subsequently cells were collected, washed, permeabilized and stained by adding fluorochrome solution (HFS –solution containing PI 50µg/ ml). After 30 min incubation at 4°C in the dark, cells were analysed by flow cytometry (FACS Canto II, BD Biosciences, Franklin Lakes, NJ, USA). Cell cycle analysis and subG1 cells were determined with FlowJo 7.6 analysis software.

Immunoblotting

Proteins were separated by SDS-PAGE and transferred onto PVDF membranes by tank blotting. The following primary antibodies were used: CASP3 (sc-7148, *Santa Cruz*), CASP8 (#9746, *Cell Signaling*), EMG1 (SAB1406991, *Sigma-Aldrich*), NOC4L (HPA053424, Sigma Aldrich) NOP14 (HPA039596, *Sigma-Aldrich*), PARP (#9542, *Cell Signaling*). Proteins were detected by chemiluminescence detection using ECL solution and developing membranes on the ChemiDoc Touch (*Bio-Rad, Hercules, CA, USA*) imaging system. Loading control was either performed by adding 0.5% TCE (2,2,2- Trichloroethanol, Sigma-Aldrich) to the PAGE gels according to the TGX Stain-Free Gels system (*Bio-Rad, Hercules, CA, USA*) or expression levels of Actin (A2066, *Sigma-Aldrich*) were used .

Immunostaining

Cells were seeded into ibidiTreat 8-well µ-slides (*ibidi GmbH, Germany*), washed, fixed with 4% paraformaldehyde and permeabilized with 0.1% TritonX-100. After blocking, cells were incubated with anti-NOP14 and anti-EMG1 antibody and the appropriate secondary antibodies: Alexa Fluor 488 goat anti mouse and Alexa Fluor 647 chicken anti rabbit (*Invitrogen, Carlsbad, CA, USA*). Nuclei were stained using 5µg/ml Hoechst33342. Confocal microscopy was performed on a Leica SP8 LSM system (*Leica Microsystems, Wetzlar, Germany*).

Nuclear Run on assay

Cells were seeded into ibidiTreat 8-well µ-slides and 5mM 5-Fluorouracil (5-FU) was added for the last 60 min. Fixation and permeabilization was performed as described before. Subsequently, cells were stained with anti-BrdU antibody (B8434, *Sigma-Aldrich*) as primary antibody and Alexa Fluor 488 goat anti mouse (*Invitrogen, Carlsbad, CA, USA*) as secondary antibody. Percentages of positive cells were calculated. Treatment with actinomycin D (6 µM) served as positive control for transcriptional inhibition.

Dual-luciferase splicing assay

HeLa cells were seeded into 6-well plates and allowed to adhere overnight. Attached cells were either transfected with an intron-containing (Luc-I, #62858, Addgene) or intronless (Luc,

#62857, *Addgene*) firefly luciferase reporter and an empty vector Renilla-Luc control plasmid (pGL4.74, *Promega*). FuGene HD served as transfection reagent and was used according to manufacturer's instructions and cells were transfected for 16h. Next, transfection reagent containing medium was removed and fresh growth medium was added before stimulation with **VioA** or the general splicing inhibitor isoginkgetin for 24h. Subsequently luciferase assay was performed using Dual-Luciferase Reporter Assay System (*Promega* E1960) following the manufacturer's instructions.

Co-immunoprecipitation (co-IP); western blot based

Jurkat cells were seeded at a density of 1 x 10^5 cells/ ml and treated as indicated. Next, cells were pelleted, washed with ice-cold PBS and cell lysis was performed by incubating cells with Triton-X 100 lysis buffer for 30 min on ice. After removal of cell debris by centrifugation, protein concentration was determined and 500 µg protein per sample was mixed with 10µl precipitation antibody (NOP14: HPA039596, *Sigma-Aldrich*) and 50 µl µMACS Protein G magnetic beads. The mixture was incubated under gentle agitation at 4°C for 4h. In parallel 500 µg protein, mixed with 5 µl normal rabbit IgG1 control antibody (#2729, *Cell Signaling*) and 50 µl magnetic beads was prepared as negative control. For co-IP, the magnetic labelled NOP14 and its interacting proteins were retained on Miltenyi µColumns that were placed into the µMACS separator magnetic field and equilibrated with Triton-X 100 buffer before sample loading. After four washing steps with Triton-X 100 buffer the proteins were eluted with 1x SDS sample buffer pre-heated to 95°C and subjected to western blot.

5. Proteomics methods

Affinity-based protein profiling

For preparative labelling experiments, $6x10^6$ Jurkat cells were resuspended in 6 mL medium w/o FBS and supplemented with 6 µL of **VioA-P** in varying concentrations (stock solution in DMSO, 0.1% final DMSO concentration) or DMSO respectively and seeded into 15 cm dishes (*Sarstedt*). After incubation (37°C, 5% CO_2, 1 h), samples were subjected to UV irradiation (*Philips* TL-D BLB UV lamps, 365 nm) for 20 min while cooling on ice. Cells were detached using a cell scraper, washed with PBS (10 mL) and lysed using pre-cooled lysis buffer (1 mL, 1% (v/v) NP40, 1% (w/v) sodium deoxycholate in PBS) for 30 min on ice. Cell debris was removed by centrifugation (21,000 g, 30 min, 4°C), resulting supernatants transferred into new falcons, and protein concentration determined using the Roti®-Quant universal kit (*Carl Roth*) for BCA assay. Equal amounts of protein (360 µg) were adjusted to a final volume of 280 µL. For copper (I)-catalyzed azide-alkyne cycloaddition, samples were supplemented with trifunctional linker (100 µM, 10 mM stock solution in DMSO), THPTA ligand (500 µM, 10 mM stock solution in DMSO, *Sigma-Aldrich*) and $CuSO_4$ (1 mM, 50 mM stock solution in H_2O). Reaction was initiated by the addition of sodium ascorbate (2 mM, 100 mM stock solution in H_2O). Samples were briefly vortexed and incubated for 1h in the dark at room temperature. Cycloaddition was quenched and proteins precipitated by the addition of 4-fold excess of acetone (1.3 mL) and incubated overnight at -20°C. Next, precipitated proteins were pelleted (21,000 g, 20 min, 4°C) and supernatant discarded. For detergent removal, protein pellet was resuspended in cold methanol (500 µL) using ultrasonication (10% intensity, 10 sec, Sonopuls HD 2070 ultrasonic rod, *BANDELIN electronic GmbH & Co. KG*). Procedure was carried out twice. 0.4% (w/v) SDS in PBS (1 mL) was added to the samples, and protein pellets dissolved by sonication (10% intensity, 10 sec, Sonopuls HD 2070 ultrasonic rod, *BANDELIN electronic GmbH & Co. KG*) at room temperature. Prior to enrichment, avidin-agarose beads (50 µL, *Sigma-Aldrich*) were equilibrated by washing with 0.4% (w/v) SDS in PBS (3x1 mL). Subsequently, samples were added to equilibrated avidin-agarose beads and incubated under continuous inverting for 1 h at room temperature. Removal of unspecific binding proteins was accomplished by washing the beads with 0.4% (w/v) SDS in PBS (3x 1 mL, 400 g, 2 min) and PBS (5x 1 mL, 400 g, 2 min). Next, beads were resuspended in X-buffer (200 µL, 7 M urea, 2 M thiourea in 20 mM HEPES buffer pH 7.5) and proteins reduced by the addition of DTT (1 mM, 1 M stock in ddH_2O) and incubated under gentle mixing (25°C, 45 min, 450 rpm). Alkylation of proteins was achieved by the addition of IAA (5.5 mM, 550 mM stock in ddH_2O)

and incubated under gentle mixing (25°C, 30 min, 450 rpm). Alkylation reaction was stopped by the addition of DTT (4 mM, 1 M stock in ddH_2O) and incubated under gentle mixing (25°C, 30 min, 450 rpm). Proteins were predigested with LysC (3.33 µg/mL, 0.5 mg/mL stock in ddH_20; *Wacko*) under gentle mixing (25°C, 2 h, 450 rpm). TEAB buffer (600 µL, 50mM, *Sigma-Aldrich*) was added, and samples further digested with trypsin (2 µL, 0.5 mg/mL stock, sequencing grade, modified; *Promega*) overnight under mixing (37°C, 800 rpm). Digest was stopped the following day by the addition of formic acid (0.1% final concentration; *Sigma-Aldrich*) and peptides desalted using Sep-Pak C18 1 cc Vac cartridges (*Waters*) and the following procedure: Resin was washed with MeCN (1 mL) and elution buffer (80% MeCN, 0.5% FA) prior to equilibration with 0.1% TFA (3 mL). Peptide solutions were loaded to the cartridges and bound peptides washed with 0.1% TFA (3 mL) and 0.5% FA (0.5 mL) and finally eluted with elution buffer (0.75 mL). Peptides were dried using a centrifugal vacuum concentrator (*Eppendorf*) and stored at -80°C. Prior to LC-MS/MS analysis dried peptide samples were reconstituted in 1% FA in ddH_2O (40 µL), sonicated for 10 min and filtered using 0.22 µm Ultrafree-MC® centrifugal filters (*Merck*, UFC30GVNB), preequilibrated with 300 µL 1% FA in ddH_2O and transferred into LC-MS vials. Experiments were carried out in triplicates.

Thermal protein profiling (TPP)

In situ thermal proteome profiling experiments were carried out as reported previously[7] with minor modifications. $6x10^7$ Jurkat cells were washed with PBS, resuspended in medium w/o FBS (15 mL) and supplemented with **VioA** (1µM; 1 mM stock solution in DMSO, 0.1% final concentration of DMSO) or DMSO respectively and transferred into T75-flasks (*Sarstedt*). After incubation (37°C, 5% CO_2, 1 h) cells were pelleted (800 g, 6 min, rt), supernatant discarded, cells resuspended in PBS (15 mL) and centrifuged again. Resulting cell pellets were taken up in PBS (1 mL) and cell suspension distributed into 0.2-mL PCR tubes (*Kisker Biotech*, $6x10^6$ Mio cells per tube) and kept at room temperature prior to heat treatment. **VioA** and DMSO treated samples were heated in parallel for 3 min. to the respective temperature (37°C, 41.2°C, 44°C, 48°C, 53.1°C, 56.3°C, 59.3°C, 62.5°C and 67.1°C) in a PCR cycler (*Techne*), incubated for 3 min. at room temperature, and subsequently snap-frozen in liquid nitrogen and stored at -80°C. Cell lysis was accomplished using four freeze-thaw cycles (incubation for 30 seconds at 25°C, followed by snap-freezing in liquid nitrogen). Subsequently, PBS (50 µL) was added and the samples subjected to ultracentrifugation (100,000 g, 20 min, 4°C). 100 µL of the resulting supernatant was transferred into new 1.5 mL LoBind tubes (*Eppendorf*). For protein

amount normalization, protein concentration of the two lowest temperature point samples (37°C and 41°C) were determined with a sample aliquot (10 µL) using Roti®-Quant universal kit (*Carl Roth*). Proteins were precipitated by adding acetone (400 µL) and incubated overnight (-20°C). Next, precipitated proteins were pelleted (21,000 g, 20 min., 4°C), supernatant discarded and protein pellet resuspended by sonication (10% intensity, 10 sec, Sonopuls HD 2070 ultrasonic rod, *BANDELIN electronic GmbH & Co. KG*) in pre-chilled methanol (500 µL). Protein pellet washing step was carried out twice. Subsequently, proteins were resuspended in X-buffer (300 µL, 7 M urea, 2 M thiourea in 20 mM Hepes buffer pH 7.5) by sonication (procedure as described above). Proteins were reduced with DTT (1 mM, 1 M stock in ddH_2O) and incubated under gentle mixing (25°C, 45 min, 450 rpm). Alkylation of proteins was achieved by the addition of iodoacetamide (5.5 mM, 550 mM stock in ddH_2O) and incubated under gentle mixing (25°C, 30 min, 450 rpm). Alkylation reaction was stopped by the addition of DTT (4 mM, 1 M stock in ddH_2O) and incubated under gentle mixing (25°C, 30 min, 450 rpm). Proteins were predigested with LysC (3.33 µg/mL, 0.5 mg/mL stock in ddH_2O; *Wacko*) under gentle mixing (25°C, 2 h, 450 rpm). TEAB buffer (900 µL, 50 mM, *Sigma-Aldrich*) was added, and samples further digested with trypsin (4 µL, 0.5 mg/mL stock, sequencing grade, modified; *Promega*) overnight under mixing (37°C, 800 rpm). Digest was stopped the following day by the addition of formic acid (0.1% final concentration; *Sigma-Aldrich*) and peptides desalted using Sep-Pak C18 1 cc Vac cartridges (*Waters*) and the following procedure: Resin was washed with MeCN (1 mL) and elution buffer (80% MeCN, 0.5% FA) prior to equilibration with 0.1% TFA (3 mL). Peptide solutions were loaded to the cartridges and bound peptides washed with 0.1% TFA (3 mL) and 0.5% FA (0.5 mL) and finally eluted with elution buffer (0.75 mL). Peptides were dried using a centrifugal vacuum concentrator (*Eppendorf*) and stored at -80°C. Peptides were reconstituted in elution buffer and a volume of each sample corresponding to 25 µg protein in the two lowest temperature points transferred into new 1.5 mL LoBind tubes. Samples were dried and taken up in TMT buffer 1 (10 µL, 10% MeCN 90% 200 mM TEAB buffer) and incubated under gentle mixing (25°C, 15 min, 400 rpm)**.** Additional TMT buffer 1 (25 µL) was added to each sample. TMT isobaric labels (TMT10plex™ isobaric Labels Reagent Set 1x 0.8 mg, *Thermo Fisher Scientific*) were allowed to gain room temperature and taken up in MeCN (41 µL). 10 µL of each label solution was added to a sample according to applied temperature point (see Table S6) and incubated under gentle mixing (25°C, 1 h, 400 rpm). Reaction was stopped by the addition of 5% (v/v) hydroxylamine solution (5 µL, *Sigma-Aldrich*) and incubated under gentle mixing (25°C, 15 min, 400 rpm). Labeled peptides were combined into a single sample per experiment, TMT

solution 2 (100 µL, 60% (v/v) 200 mM TEAB buffer 40% (v/v) MeCN) added and samples dried using a centrifugal vacuum concentrator. Dried labeled peptides were reconstituted in 0.5% formic acid (200 µL) and desalted using C18 StageTips (eight layers; Empore disk-C18; 47 mm; *Agilent Technologies*) and the following procedure: layers were washed with MeOH (90 µL), elution buffer (200 µL) and 0.5% FA (600 µL). Samples were loaded on stage tips (500g, 2 min) and bound peptides washed with 0.5% formic acid (600 µL) and eluted with elution buffer (300 µL). Eluted peptides were dried in a centrifugal vacuum concentrator. For HILIC fractionation, samples were reconstituted in HILIC buffer A (110 µL, 95% MeCN 5% H_2O and 0.1% TFA), sonicated, vortexed and centrifuged (21,000 x g, 10 min, 4°C). Peptide fractionation was carried out using an UltiMate 3000 HPLC system (*Dionex*) equipped with an YMC-Pack PVA-Sil column (5 µm, 150 x 2.1 mm, 120 Å, *YMC Europe GmbH*). Gradient elution was carried out with 95% MeCN 5% H_2O and 0.1% TFA (A) and 95% H_2O 5% MeCN and 0.1% TFA (B). 100 µL sample were injected and separated using a 62.5 min gradient (7.5 min 0% B, 50 min to 30% B, 3.5 min to 50% B and 2.5 min to 100% B) at a flow rate of 0.2 mL/min, followed by a washing and column re-equilibration step (12.5 min 100% B, 0.5 min to 0% B and 22.5 min 0% B). During separation, an on-line UV detector set at 215 nm was utilized to monitor peptide mixture elution. Fractions were collected into a 96-well plate (*Eppendorf*) and resulting fractions were pooled into 10 greater fractions. Fractions were dried in a centrifugal vacuum concentrator and fractions 2-9 subjected to LC-MS/MS analysis. Prior to LC-MS/MS analysis dried peptide samples were reconstituted in 1% FA in ddH_2O (10 µL), sonicated for 10 min and filtered using 0.22 µm Ultrafree-MC® centrifugal filters (*Merck*, UFC30GVNB), preequilibrated with 300 µL 1% FA in ddH_2O and transferred into LC-MS vials. The experiment was carried out in duplicates.

MS-based co-Immunoprecipitation

For MS-based co-Immunoprecipitation experiments $4x10^6$ cells were plated out on 6 cm dishes (*Sarstedt*) in 8 mL media supplemented with **VioA** (10 nM, 10 µM stock solution in DMSO, 0.1% final concentration of DMSO) or DMSO respectively. After incubation (37°C, 5% CO_2, 24 h) 37°C, 5% CO_2, cells were counted and readjusted to a number of $4x10^6$ cells per dish. Cells were subsequently, pelleted (800 x g, 6 min, rt), supernatant discarded, and cells washed in PBS (2 mL) twice. For *in situ* cellular cross-linking cells were resuspended in PBS (1 mL) supplemented with DSSO crosslinker (2 mM, 100 mM stock solution in DMSO, 2% final concentration of DMSO, synthesized as described previously[10]). After incubation (37°C, 5% CO_2, 1 h) cells were pelleted (600 g, 5 min, 4°C), supernatant discarded, and remaining DSSO

crosslinker quenched by resuspending cells in cold tris buffer (1 mL, 50 mM Tris-HCl, pH = 8.0). Cells were pelleted (600 g, 5 min, 4°C), resuspended in cold PBS (1 mL), pelleted and supernatant discarded. For cell lysis, cells were taken up in IP lysis buffer (500 µL, 50 mM Tris-HCl, 150 mM NaCl, 1 mM $MgCl_2$, 5% (v/v) glycerol, 1% (v/v) NP-40, pH = 7.4) and incubated for 30 min on ice. Cell lysate was cleared from debris (21,000 g, 20 min, 4°C), protein concentrations determined using Roti®-Quant universal kit (*Carl Roth*) and samples normalized to a total protein amount of 500 µg per sample (protein concentration 1 µg/µL). 30 µL Protein A/G Agarose beads (*Pierce Biotechnology, Thermo Fisher Scientific*) were equilibrated with cold IP wash buffer (1 mL, 50 mM Tris-HCl, pH = 7.4, 150 mM NaCl, 1 mM $MgCl_2$, 5% (v/v) glycerol, 0.05% NP-40), centrifuged (1,000 g, 1 min, 4°C), supernatant discarded and 500 µL of sample added directly to the beads. Additionally, 10 µL of the polyclonal rabbit anti-NOP14 antibody (stock concentration 0.1 mg/mL, *Sigma-Aldrich*), was added. For determination of unspecific background binding to the beads and the antibody constant regions, 0.4 µL of isotype control rabbit mAb IgG (stock concentration 2.5 mg/mL, *Cell Signaling Technology*) was added to control samples, and all samples incubated at 4°C under constant rotation overnight. Subsequently, samples were centrifuged (1,000 g, 1 min, 4°C) and supernatant discarded. For removal of unspecific bound proteins, pelleted beads were resuspended in IP wash buffer (1 mL), centrifuged (500 g, 30 sec, 4°C), and supernatant discarded. The procedure was carried out twice, and twice again with IP basic buffer (50 mM Tris-HCl, pH = 7.4, 150 mM NaCl, 5% (v/v) glycerol) for detergent removal. Samples were reduced and digested by the addition of IP elution buffer I (25 µL, 50 mM Tris-HCl, pH = 8.0; 5 ng/µL Trypsin (500 ng/µL stock solution, sequencing grade, modified, *Promega*), 2 M Urea, 1 mM DTT (500 mM stock solution in H_2O)). Samples were incubated (30 min, 600 rpm, rt) prior to addition of IP elution buffer II (100 µL; 50 mM Tris-HCl; pH = 8.0; 2 M Urea; 5 mM IAA (500 mM stock solution in H_2O)), followed by incubation at 37°C and 600 rpm overnight. Tryptic digest was stopped by the addition of FA (1% final concentration). Samples were desalted using C18-stage tips (double layer; Empore disk-C18; 47 mm; *Agilent Technologies*) using the following procedure: Equilibration with MeOH (70 µL), followed by washing with 0.5% FA (3x 70 µL), sample load and a washing step (3x 70 µL, 0.5% FA). Finally, peptides were eluted with elution buffer (3x 30 µL; 80% MeCN, 0.5% FA). Samples were dried using a centrifugal vacuum concentrator (*Eppendorf*). Prior to LC-MS/MS analysis dried peptide samples were reconstituted in 1% FA in ddH_2O (26 µL), sonicated for 10 min and filtered using 0.22 µm Ultrafree-MC® centrifugal filters (*Merck*, UFC30GVNB), preequilibrated with

300 µL 1% FA in ddH_2O and transferred into LC-MS vials. The experiment was carried out in quadruplicates.

Mass spectrometry for Affinity-based protein profiling

Samples were separated and analyzed with an Ultimate 3000 Nano HPLC system (*Thermo Fisher Scientific*) equipped with an Acclaim C18 PepMap100 75µm ID x 2 cm trap column and an Acclaim PepMap C18 RSLC 75 µm ID x 50 cm separation column in an EASY-Spray™ setting, coupled to an Q Exactive Plus (*Thermo Fisher Scientific*). For peptide separation, samples were loaded on the trap column and washed for 10 min with 0.1% TFA in ddH_2O at a flow rate of 5 µL/min. Subsequently, peptides were transferred to the analytical column for peptide separation and separated using the following 132 min gradient (Buffer A: H_2O + 0.1% FA; B: MeCN + 0.1% FA) with a flow rate of 300 nL/min.: in 7 min to 5% B, in 105 min from 5% to 22%, in 10 min from 22 to 35% and in another 10 min to 90% B. Separation gradient was followed by a column washing step using 90% B for 10 min and subsequent column re-equilibration with 5% B for 5 min. Peptides were ionized using an EASY-Spray™ source with variable voltage and a capillary temperature of 275°C. Q Exactive Plus was operated in a TopN data dependent mode of 12. MS full scans (scan range 375 – 1,500 m/z) was performed in the orbitrap at a resolution of R = 140,000 (at 200 m/z) and an automatic gain control (AGC) ion target of 3.0e6 with a maximum injection time of 80 ms and RF Lens amplitude set to 60%. Peptides with charge states of 2 – 7 and an intensity higher than 1e4 were isolated in the quadrupole using a window of 1.6 m/z and subjected to higher-energy collisional dissociation (HCD; HCD collision Energy set to 27%). Dynamic exclusion was set to 60s. MS^2 scans were recorded in the orbitrap at a resolution of R = 17,500, with an AGC target set to 1e3 and a maximum injection time of 100 ms.

Mass spectrometry for MS-based co-Immunoprecipitation

Samples were separated and analyzed with an Ultimate 3000 Nano HPLC system (Thermo Scientific) equipped with an Acclaim C18 PepMap100 75 µm ID x 2 cm trap column and an Acclaim PepMap C18 RSLC 75 µm ID x 50 cm separation column in an EASY-Spray™ setting, coupled to an LTQ Orbitrap Fusion (Thermo Scientific).

For peptide separation, samples were loaded on the trap column and washed for 10 min with 0.1% TFA in ddH_2O at a flow rate of 5 µL/min. Subsequently, peptides were transferred to the

analytical column for peptide separation and separated using the following 132 min gradient (Buffer A: H2O + 0.1% FA; B: MeCN + 0.1% FA) with a flowrate of 300 nL/min.: 7 min at 5%B, in 105 min from 5% to 22%, in 10 min from 22 to 35% and in another 10 min to 90% B. Separation gradient was followed by a column washing step using 90% B for 10 min and subsequent column re-equilibration with 5% B for 5 min. Peptides were ionized using an EASY-Spray™ source with variable voltage and a capillary temperature of 275°C. Orbitrap Fusion was operated in top speed mode with 3 s cycle time. Internal calibration was performed by using the ion signal of fluoranthene cations (EASY-ETD/IC source). MS full scans (300 – 1500 m/z) were performed in the orbitrap at a resolution of R = 120,000 (at 200 m/z) with an automatic gain control (AGC) ion target of 2e5 and a maximum injection time of 80 ms and RF Lens amplitude set to 60%. Monoisotopic precursor selection was enabeled and dynamic exclusion set to 60 s. Most intense peptides with charge states of 2 – 7 and an intensity higher than 5e3 were isolated in the quadrupole using a window of 1.6 m/z and subjected to higher-energy collisional dissociation (HCD; HCD collision energy set to 30%). MS^2 ions were collected in the iontrap to a target of 1e4 for maximum injection time of 100 ms with "inject ions for all available parallelizable time" enabeled.

Mass spectrometry for thermal proteome profiling

Samples were analyzed by LC-MS/MS using an Ultimate 3000 Nano HPLC system (*Thermo Fisher Scientific*) equipped with an Acclaim C18 PepMap100 75µm ID x 2 cm trap column and an Acclaim PepMap C18 RSLC 75 µm ID x 50 cm separation column in an EASY-Spray™ setting, coupled to an LTQ Orbitrap Fusion (*Thermo Fisher Scientific*).
Prior to separation, samples (3 µL) were loaded on the trap column and washed for 10 min with 0.1% TFA in ddH_2O at a flow rate of 5 µL/min. Subsequently, peptides were transferred to the analytical column for peptide separation and separated using the following 120 min gradient (Buffer A: H_2O + 0.1% FA; B: MeCN + 0.1% FA) with a flow rate of 300 nL/min.: 10 min 5% B, in 50 min from 5% to 22%, in 60 min from 22 to 35% B. Separation gradient was followed by a column washing step using 90% B for 10 min and subsequent column re-equilibration with 5% B for 5 min. Peptides were ionized using an EASY-Spray™ source (Spray Voltage = 2.2 kV) and a capillary temperature of 275°C. LTQ Orbitrap Fusion was operated in a top speed data-dependent mode with 3 s cycle time. Internal calibration was performed by using the ion signal of fluoranthene cations (EASY-ETD/IC source). MS full scans (scan range 375 – 1,500 m/z) were performed in the orbitrap at a resolution of R =

120,000 (at 200 m/z) with an automatic gain control (AGC) ion target of 4.0e5 with a maximum injection time of 50 ms and RF Lens amplitude set to 60%. Dynamic exclusion was set to 60 s. Most intense peptides with charge states of 2 – 7 and an intensity higher than 5e4 were isolated in the quadrupole using a window of 1.0 m/z and subjected to higher-energy collisional dissociation (HCD; HCD collision energy set to 35%). MS^2 ions were collected in the iontrap to a target of 1e5 for maximum injection time of 105 ms.

6. Targeted metabolic assay

Sample preparation

Several flasks of cultured Jurkat cells were pooled and centrifuged (800g, 10 min, rt). Resulting cell pellet was washed with cold PBS (80 mL) and pelleted again. Cells were lysed by resuspending cell pellet in cold lysis buffer (1 mL, 1% (v/v) NP40, 1% (w/v) sodium deoxycholate in PBS) and incubation for 30 min on ice. Subsequently, cell debris was removed by centrifugation (21,000 g, 15 min, 4°C) and resulting supernatants transferred into new falcons and protein concentration determined using the Roti®-Quant universal kit (*Carl Roth*) for BCA assay. Protein concentration was adjusted to 1 mg/mL by diluting samples with lysis buffer and stored at -80°C until further use. For time-dependent degradation analysis of **VioA-P**, cell lysate (400 µL) was incubated with **VioA-P** (50 µM, 50 mM stock in DMSO, 0.1% final concentration of DMSO) under gentle mixing (800 rpm, 37°C). At the respective time points (0, 1, 2, 4, 6, 8 and 24 h) an aliquot (50 µL) of the solution was transferred into a new Lobind tube and proteins precipitated by adding MeCN (200 µL) and incubated overnight (-20°). Next, precipitated proteins were pelleted (21,000 g, 30 min, 4°C) and supernatants transferred into LC-MS vials. Experiment was carried out in triplicates.

Mass spectrometry for targeted metabolic assay

Metabolic profiling and MS/MS analysis was carried out on a Ultimate^{TM} 3000 RSLC system (*Thermo Scientific*) coupled to an LTQ Orbitrap XL mass spectrometer (*Thermo Scientific*). Chromatographic separation was carried out using an Accucore C18 aQ (150 x 2.1 mm, 2.6 µm, *Thermo Scientific*) column at 40°C and H_2O + 0.1% FA (A) and MeCN + 0.1% FA (B). Following gradient was applied for sample elution (flow rate: 0.5 mL/min): 3 min pre-equilibration with 2% B, 0.5 min 2% B, in 9.5 min from 2% to 100% B, 3 min at 100% B, in 0.5 min from 100% B to 2% B, followed by column re-equilibration at 2% B for 2.5 min. Mass spectrometric measurements were accomplished in positive ion mode (HESI-II source, *Thermo Scientific*) with following source parameters: capillary voltage 4.2 kV, tube lens 80 V, sheath gas 60 L/h, aux gas 10 L/h, capillary voltage 48 V and capillary temperature 350°C. Full scan measurements were recorded between 50 – 1500 m/z in centroid mode at 60,000 resolution in the orbitrap. Ions of interest (**VioA** m/z 863.42, **VioA-P** m/z 997.48) were isolated in single ion monitoring (SIM) profile mode and used for relative quantification (SIM width 10 m/z) at a

resolution of 30,000 in the orbitrap. Prior to measurement 10 pooled QC samples were injected (injection volume for all samples 5 µL) to equilibrate the column. Sample order was randomized in order to take metabolic degradation over time into account.

7. Bioinformatics

Analysis of Affinity-based protein profiling and MS-based co-IP experiments

MS raw data was analyzed using MaxQuant[11] software (version 1.6.2.10) with Andromeda[12] search engine (searches were carried out against Uniprot database for Homo sapiens (taxon identifier: 9606, downloaded on 15.10.2018, canonical). Carbamidomethlyation of cysteines (C) was set as fixed modifications and oxidation of methionines (M) and acetylation of N-termini as variable modifications. Trypsin (without N-terminal cleavage to proline) was set as proteolytic enzyme with a maximum of two allowed missed cleavages. For main search, precursor mass tolerance was set to 4.5 ppm and fragment mass tolerance to 0.5 Da. Label free quantification (LFQ) mode was activated with a LFQ minimum ratio count of 1. Second peptide identification was enabled, and false discovery rate (FDR) determination carried out by applying a decoy database and thresholds were set to 1% FDR at peptide-spectrum match and at protein levels and "match between runs" (0.7 min match and 20 min alignment time windows) option was enabled. Normalized LFQ intensities extracted from the MaxQuant result table proteinGroups.txt were further analyzed with Perseus[13] software (version 1.6.3.2). Prior to analysis, putative contaminants, reverse hits and only identified by site hits were removed. Normalized LFQ intensities were $\log_2$ transformed and proteins with two valid values for AfBPP data and three valid values for co-IP data in at least one group were used for missing value imputation from normal distribution (width 0.3, downshift 1.8, total matrix). Two-sample Students's *t*-test including Benjamini-Hochberg multiple testing correction (FDR = 0.05) was performed. Proteins with an enrichment factor of 2 ($\log_2(x) = 1$) and -$\log_{10}$ *t*-test *p*-value of 1.3 for AfBPP data and an enrichment factor of 4 ($\log_2(x) = 2$) for co-IP data and -$\log_{10}$ *t*-test *p*-value of 1.3 were considered as significantly enriched proteins.

Data analysis for thermal proteome profiling experiments

Recorded raw files were processed using MaxQuant[11] software (version 1.6.2.10) and MS/MS spectra were searched against the Uniprot FASTA database for *homo sapiens* (taxon identifier: 9606, downloaded on 15.10.2018, canonical). Trypsin (without proline) was set as proteolytic enzyme with a maximum of two missed cleavages allowed. Fractions were assigned according to HILIC fractionation. Group-specific parameters were set to "Reporter ion MS2" with 10plex TMT isobaric labels for N-terminal and lysine residue modification selected. Reporter mass

tolerance was set to 0.003 Da and Filter by PIF activated (min. reporter PIF set to 0.75). Ion isotopic distribution was set for each TMT label (N-terminal and lysine) as stated in Table S5 for the use of isotope correction.

Cysteine carbamidomethylation was set as fixed modification and methionine oxidation as well as N-terminal protein acetylation as variable modifications with a max. number of modifications per peptide set to 5. The second peptide identification and “match between runs” (0.7 min match time window, 20 min alignment time window) was enabled. False discovery rate determination was carried out using a decoy database and thresholds were set to 1% FDR at peptide-spectrum match and at protein levels. Protein quantification was carried out with label minimum ratio count set to 2 and peptides for quantification set to “unique and razor”. Remaining parameters were utilized as given in the default settings.

Downstream data analysis of corrected reporter intensities calculated by MaxQuant in the protein groups table were further used for the determination of melting curves and T_m shifts, which was performed with R (version 3.5.1 “Feather Spray”) and the TPP[7] R package (version 3.0.3). Intensities of channel TMT^{10}-128C were excluded from MaxQuant outputfile as TMT label was not applied prior to downstream analysis. Corrected intensities were normalized to the lowest applied temperature TMT channel. Data analysis was carried out as stated by the authors with minor changes (`fcColumn = c(6,8,9)`).

Prior to visualization of obtained output files, data were filtered using the following criteria[7, 14]:

- $R^2 > 0.8$ for fitted curves for DMSO and **VioA** treatment
- Plateau of < 0.3 for DMSO curves
- Steepest slope of protein melting curves in paired set of DMSO and **VioA** treated conditions < -0.06.
- Melting point difference for each protein between both DMSO replicates < 1.5°C.

Following additional criteria were applied for hit identification[7, 14]:

- One of the *p* values for the two replicate experiments is < 0.1 and the other is < 0.05
- Melting point shifts for both paired replicates (**VioA** vs DMSO) have the same direction
- Melting point difference VioA vs DMSO > DMSO 1 vs DMSO 2

Data visualization was carried out using Graphpad Prism 8.

Data analysis for targeted metabolic assay

Raw data was processed with Xcalibur Quan Browser (*Thermo Scientific*) using Genesis algorithm (Smoothing points:15; S/N threshold: 0.5, Highest peak detection mode) and manual integration mode. Intensities of the corresponding ions were extracted and visualized using Graphpad Prism 8.

8. Chemical Synthesis

General remarks

Chemical reagents and solvents used for chemical synthesis were purchased in reagent grade or higher purity from *Alfa-Aesar, AppliChem, Acros Organics, Sigma-Aldrich, TCI Europe* or *Merck* and were used without further purification. 2-(3-But-3-ynyl-3H-diazirin-3-yl)-ethanol was purchased from *Ark Pharm Inc*.

Flash column chromatography as performed on silica gel (Geduran Si 60, 40-63 µm, *Merck*), and elution solvents distilled pior to use. Analytical thin-layer chromatography was performed on aluminium-baked TLC Silica gel 60 F254 plates (*Merck*) and analytes visualized by UV detection at 254 nm or stained via aq. $KMNO_4$. Synthesized compounds were dissolved in $CDCl_3$ (*Sigma-Aldrich*) for NMR measurements.

LC-ESI-HR-MS analysis of the compounds was carried out using an Thermo Finnigan LTQ FT-ICR (*Thermo Finnigan*) mass spectrometer (R = 100,000, profile mode), equipped with a Dionex Ultimate 3000 HPLC (*Dionex*). HPLC separation was accomplished with a XBridge C18 column (3.5 µm, 4.6 x 100 mm, *Waters*) at a column temperature set to 30°C. Mobile phases used for separation were 0.1% (v/v) formic acid in H_2O (*Sigma-Aldrich*, HPLC-grade) (A) and 90:10 MeCN:H_2O (*Sigma-Aldrich*, HPLC-grade) and 0.1% (v/v) formic acid (B). Gradient (flowrate 1.1 mL/min) applied: in 10 min from 20 to 98% B, kept for 4 min at 98% prior to column equilibration at 20% B for 4 min.
Fragmentation spectra of **VioA** and **VioA-P** were recorded on an LTQ Orbitrap XL (R = 30,000) in positive mode. Molecules were fragmented using normalized collision energy (NCE) = 35 with wideband activation switched on.

Synthesis of 2-(3-(but-3-yn-1-yl)-3H-diazirin-3-yl)acetic acid (**Minimalist photocrosslinker acid**)

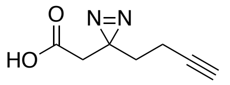

$C_7H_8N_2O_2$

152.15 g/mol

To a stirred solution of 2-(3-But-3-ynyl-3H-diazirin-3-yl)-ethanol (99 mg, 0.73 mmol, 1 eq.) in acetone (6 mL) at 0°C Jones reagent (2 M CrO_3 in H_2SO_4 (aq), 1.43 mL, 2.9 mmol , 4 eq.)was added dropwise. The reaction solution was allowed to gain room temperature and stirred for 2 h. Subsequently, the reaction was quenched with 70% isopropanol (10 mL) and filtrated over kieselgur. The filter cake was washed with acetone (3x10 mL) and the filtrate dried over $MgSO_4$, concentrated *in vacuo* and purified using flash chromatography on silica gel (hexane/ethyl acetate 4:1 + 2% acetic acid) to give minimalist photocrosslinker acid (88 mg, 0.57 mmol, 79%) as a yellowish oil.

Spectral data are consistent with those published previously.[6]

TLC (Hex/EtOAc = 4/1 + 2% acetic acid): R_f = 0.32 [UV | KMnO4]. **^{1}H NMR** (400 MHz, $CDCl_3$): δ [ppm] = 10.94 (s, 1H), 2.40 (s, 2H), 2.05 (td, *J* = 7.3, 2.6 Hz, 2H), 2.00 (t, *J* = 2.6 Hz, 1H), 1. (t, *J* = 7.3 Hz, 2H). **^{13}C NMR** (100 MHz, $CDCl_3$): δ [ppm] = 175.9, 82.5, 69.7, 39.6, 32.0, 25.3, 13.3. **ESI-HR-MS** (m/z): 303.10979 $[2M-H]^-$ calc. 303.11320

Synthesis of **VioA-P**

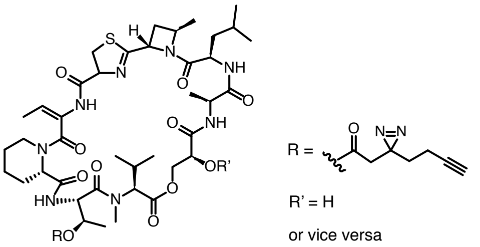

$C_{47}H_{68}N_{10}O_{12}S$

997.18 g/mol

A solution of minimalist photocrosslinker acid (7.76 mg, 51.02 µmol, 2 eq.), DMAP (156.1 µg, 1.28 µmol, 5 mol%.), and DCC (10.53 mg, 51.02 µmol, 2 eq.) in DCM (160 µL) was cooled to 0°C. **VioA** (22 mg, 25.51 µmol, 1 eq.) was dissolved in dichloromethane (200 µL) and added slowly to the solution. The reaction mixture was stirred for 4 h at 0°C and was allowed to gain room temperature overnight. Next, the solution was concentrated *in vacuo* and purified by silica gel chromatography using EtOAc/MeOH 9:1 to give **VioA-P** (7.6 mg, 7.62 µmol, 30%) as a colorless solid.

TLC (EtOAc/MeOH = 9/1): R_f = 0.37 [UV | KMnO4]. **ESI-HR-MS** positive mode (m/z): 997.48034 $[M+H]^+$ (calc. 997.48116) **HPLC analysis** A = 100% H_2O 0.1% FA; B = 90% MeCN 10% H_2O 0.1% FA, Gradient: T_0 : B = 20% T_{10} = 98% Retention time: 8.05 min.

9. NMR and LC-MS spectra

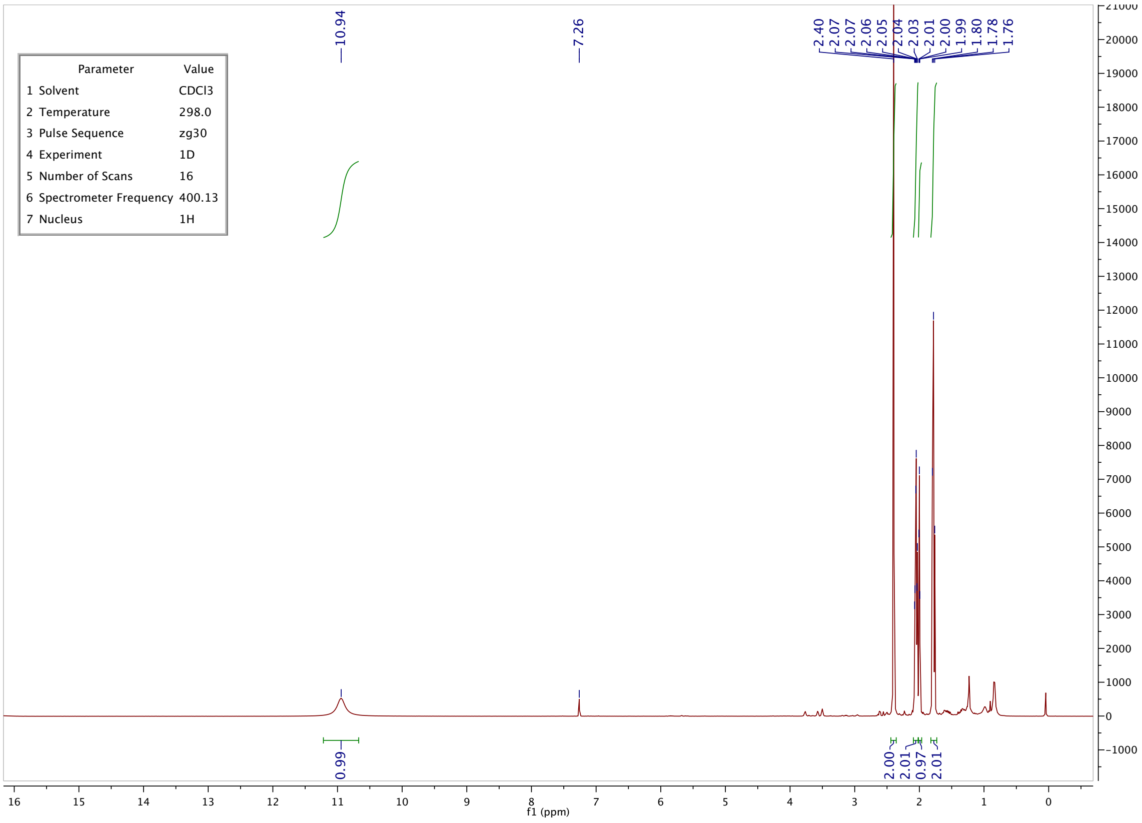

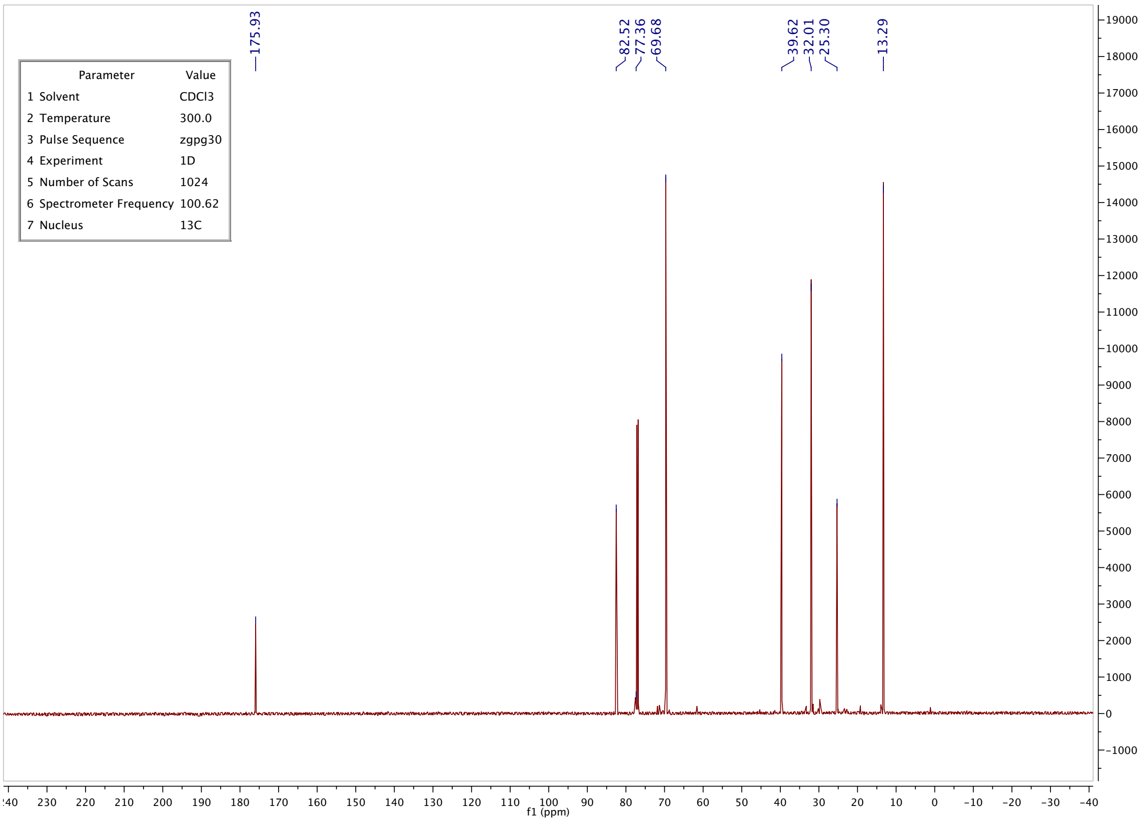

^{1}H and ^{13}C NMR spectra of 2-(3-(but-3-yn-1-yl)-3H-diazirin-3-yl)acetic acid.

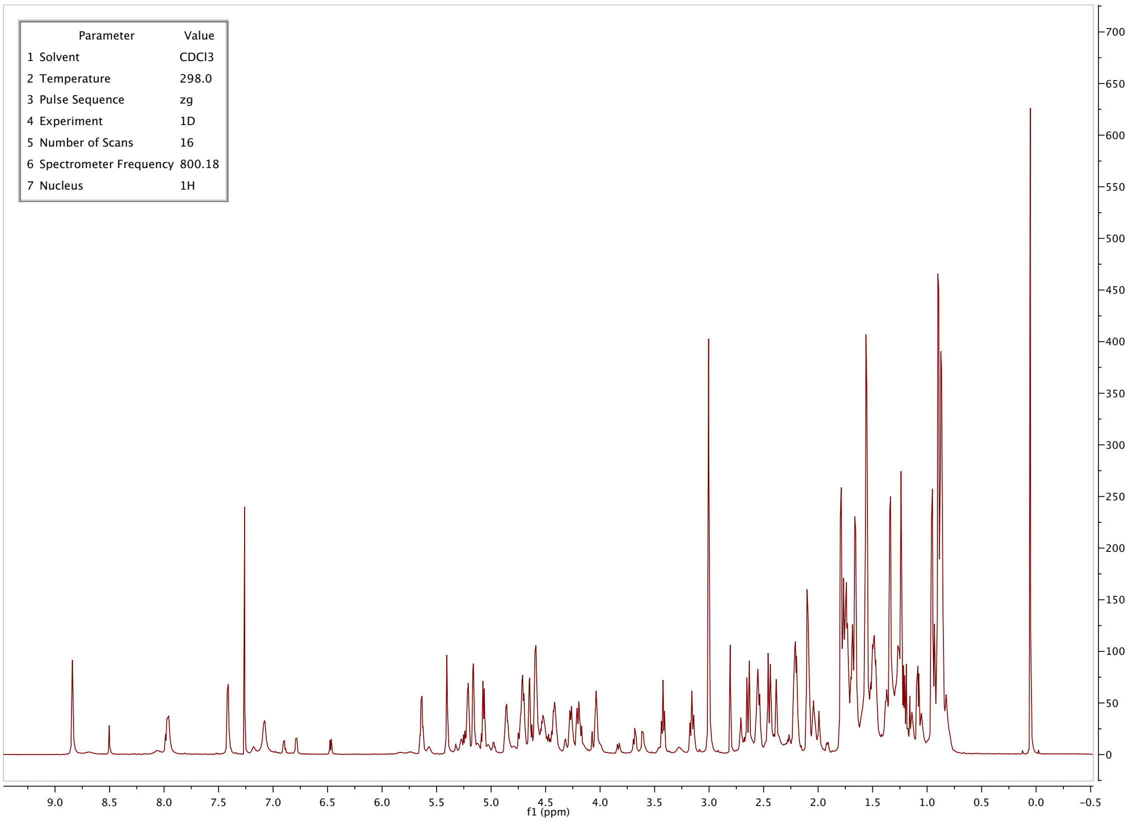

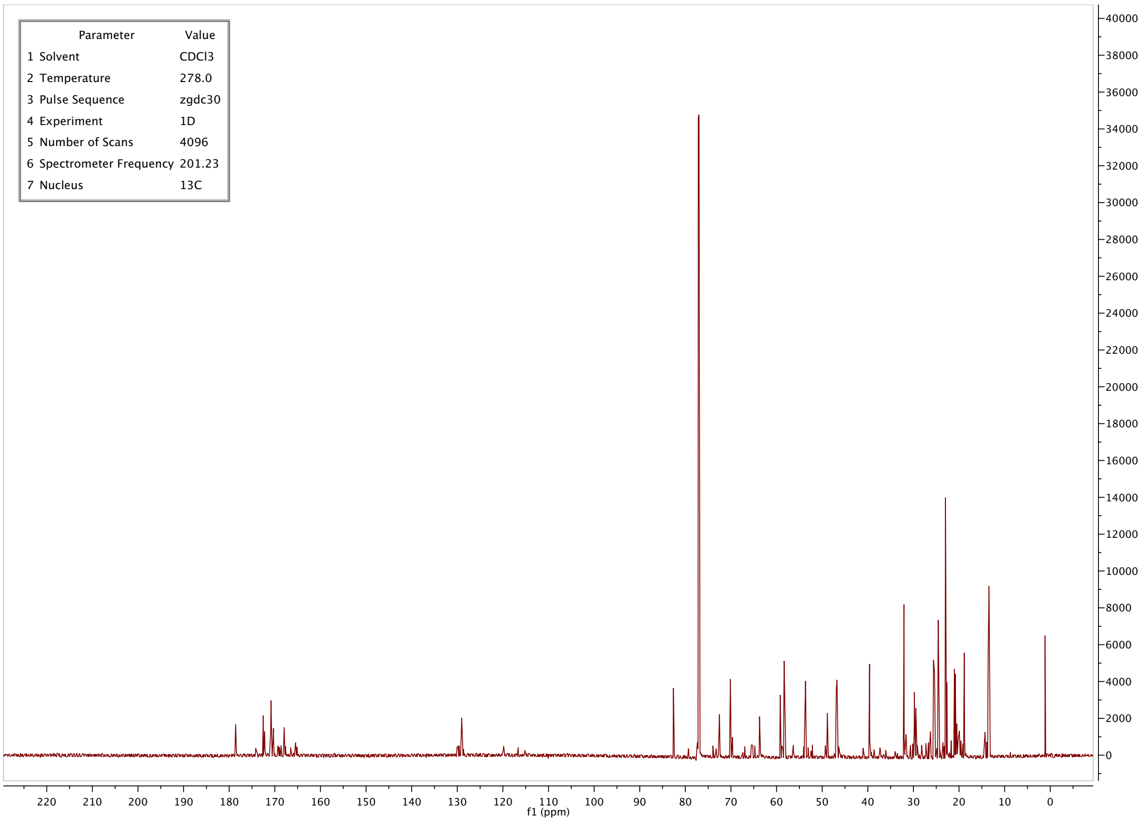

^{1}H and ^{13}C NMR spectra of **VioA-P**.

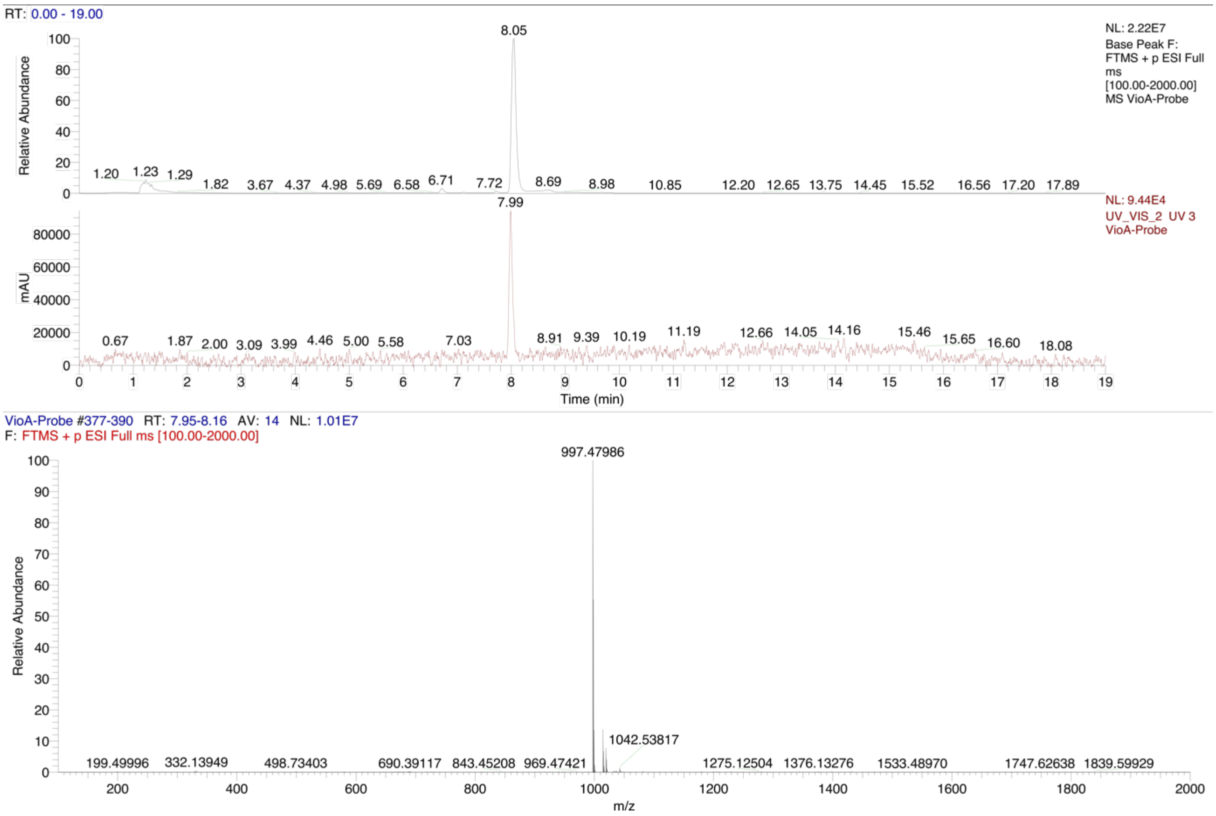

HPLC- & HR-ESI-MS spectra of **VioA-P**.

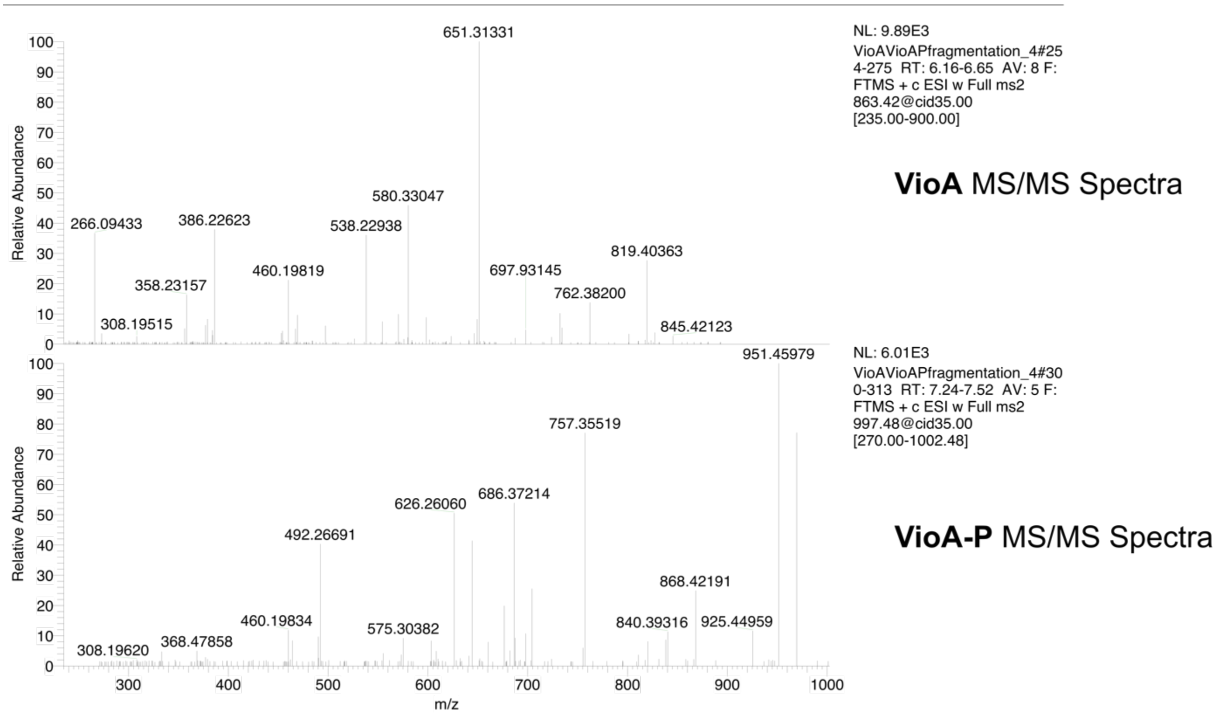

MS/MS fragmentation spectra of **VioA** and **VioA-P**.

10. Glossary

A*f*BPP	Affinity-based protein profiling
aq	Aqueous
BCA	Bicinchoninic Acid
CID	Collision-induced dissociation
Da	Dalton
DCM	Dichlormethane
ddH_2O	double-destilled water
DCC	*N,N'*-Dicyclohexylcarbodiimide
DMAP	4-Dimethylaminopyridine
DMSO	Dimethylsulfoxide
DSSO	Disuccinimidyl sulfoxide
DTT	Dithiothreitol
eq.	Equivalent(s)
EtOAc	Ethyl acetate
FA	Formic acid
HEPES	4-(2-hydroxyethyl)-1-piperazineethanesulfonic acid
HESI	Heated Electrospray Ionization
Hex	Hexane
HILIC	Hydrophilic interaction liquid chromatography
HPLC	High performance liquid chromatography
HR	High resolution
IAA	Iodoacetamide
LC	Liquid Chromatography
LysC	Endoproteinase Lys-C
MeCN	Acetonitrile
MeOH	Methanol
MS	Mass spectrometry
MS/MS	Tandem mass spectrometry
m/z	Mass to charge ratio
NMR	Nuclear magnetic resonance
PCR	Polymerase chain reaction
PBS	Phosphate buffered saline

ppm	Parts per million
QC	Quality Control
R_f	Retention factor
rpm	Rounds per minute
rt	Room temperature
SDS	Sodium dodecylsulfate
S/N	Signal to noise ratio
THPTA	Tris(3-hydroxypropyltriazolylmethyl)amine
TEAB	Tetraethylammonium bromide
TFA	Trifluoroacetic acid
TLC	Thin layer chromatography
TMT	Tandem Mass Tag
UV	Ultraviolet

11. Supplementary references

[1] Y. Perez-Riverol, A. Csordas, J. Bai, M. Bernal-Llinares, S. Hewapathirana, D. J. Kundu, A. Inuganti, J. Griss, G. Mayer, M. Eisenacher, E. Perez, J. Uszkoreit, J. Pfeuffer, T. Sachsenberg, S. Yilmaz, S. Tiwary, J. Cox, E. Audain, M. Walzer, A. F. Jarnuczak, T. Ternent, A. Brazma, J. A. Vizcaino, *Nucleic Acids Res.* **2019**, *47*, D442-D450.

[2] O. Kepp, L. Galluzzi, M. Lipinski, J. Yuan, G. Kroemer, *Nat. Rev. Drug Discov.* **2011**, *10*, 221-237.

[3] S. Vyas, E. Zaganjor, M. C. Haigis, *Cell* **2016**, *166*, 555-566.

[4] S. Xiong, T. Mu, G. Wang, X. Jiang, *Protein Cell* **2014**, *5*, 737-749.

[5] L. Galluzzi, I. Vitale, S. A. Aaronson, J. M. Abrams, D. Adam, P. Agostinis, E. S. Alnemri, L. Altucci, I. Amelio, D. W. Andrews, M. Annicchiarico-Petruzzelli, A. V. Antonov, E. Arama, E. H. Baehrecke, N. A. Barlev, N. G. Bazan, F. Bernassola, M. J. M. Bertrand, K. Bianchi, M. V. Blagosklonny, K. Blomgren, C. Borner, P. Boya, C. Brenner, M. Campanella, E. Candi, D. Carmona-Gutierrez, F. Cecconi, F. K. M. Chan, N. S. Chandel, E. H. Cheng, J. E. Chipuk, J. A. Cidlowski, A. Ciechanover, G. M. Cohen, M. Conrad, J. R. Cubillos-Ruiz, P. E. Czabotar, V. D'Angiolella, T. M. Dawson, V. L. Dawson, V. De Laurenzi, R. De Maria, K.-M. Debatin, R. J. DeBerardinis, M. Deshmukh, N. Di Daniele, F. Di Virgilio, V. M. Dixit, S. J. Dixon, C. S. Duckett, B. D. Dynlacht, W. S. El-Deiry, J. W. Elrod, G. M. Fimia, S. Fulda, A. J. García-Sáez, A. D. Garg, C. Garrido, E. Gavathiotis, P. Golstein, E. Gottlieb, D. R. Green, L. A. Greene, H. Gronemeyer, A. Gross, G. Hajnoczky, J. M. Hardwick, I. S. Harris, M. O. Hengartner, C. Hetz, H. Ichijo, M. Jäättelä, B. Joseph, P. J. Jost, P. P. Juin, W. J. Kaiser, M. Karin, T. Kaufmann, O. Kepp, A. Kimchi, R. N. Kitsis, D. J. Klionsky, R. A. Knight, S. Kumar, S. W. Lee, J. J. Lemasters, B. Levine, A. Linkermann, S. A. Lipton, R. A. Lockshin, C. López-Otín, S. W. Lowe, T. Luedde, E. Lugli, M. MacFarlane, F. Madeo, M. Malewicz, W. Malorni, G. Manic, et al., *Cell Death Differ.* **2018**, *25*, 486-541.

[6] P. Kleiner, W. Heydenreuter, M. Stahl, V. S. Korotkov, S. A. Sieber, *Angew. Chem. Int. Ed.* **2017**, *56*, 1396-1401.

[7] H. Franken, T. Mathieson, D. Childs, G. M. Sweetman, T. Werner, I. Togel, C. Doce, S. Gade, M. Bantscheff, G. Drewes, F. B. Reinhard, W. Huber, M. M. Savitski, *Nat. Protoc.* **2015**, *10*, 1567-1593.

[8] S. Ebinger, E. Z. Özdemir, C. Ziegenhain, S. Tiedt, C. Castro Alves, M. Grunert, M. Dworzak, C. Lutz, V. A. Turati, T. Enver, H.-P. Horny, K. Sotlar, S. Parekh, K. Spiekermann, W. Hiddemann, A. Schepers, B. Polzer, S. Kirsch, M. Hoffmann, B. Knapp, J. Hasenauer, H. Pfeifer, R. Panzer-Grümayer, W. Enard, O. Gires, I. Jeremias, *Cancer Cell* **2016**, *30*, 849-862.

[9] I. Nicoletti, G. Migliorati, M. C. Pagliacci, F. Grignani, C. Riccardi, *J. Immunol. Methods* **1991**, *139*, 271-279; C. Riccardi, I. Nicoletti, *Nat. Protoc.* **2006**, *1*, 1458-1461.

[10] A. Fux, V. S. Korotkov, M. Schneider, I. Antes, S. A. Sieber, *Cell Chem. Biol.* **2019**, *26*, 48-59 e47.

[11] J. Cox, M. Mann, *Nat. Biotechnol.* **2008**, *26*, 1367-1372; S. Tyanova, T. Temu, J. Cox, *Nat. Protoc.* **2016**, *11*, 2301-2319.

[12] J. Cox, N. Neuhauser, A. Michalski, R. A. Scheltema, J. V. Olsen, M. Mann, *J. Proteome Res.* **2011**, *10*, 1794-1805.

[13] S. Tyanova, T. Temu, P. Sinitcyn, A. Carlson, M. Y. Hein, T. Geiger, M. Mann, J. Cox, *Nat. Methods* **2016**, *13*, 731-740.

[14] I. Becher, T. Werner, C. Doce, E. A. Zaal, I. Togel, C. A. Khan, A. Rueger, M. Muelbaier, E. Salzer, C. R. Berkers, P. F. Fitzpatrick, M. Bantscheff, M. M. Savitski, *Nat. Chem. Biol.* **2016**, *12*, 908-910.
